# Supplementary material for: Quantitative comparison of geological data and model simulations constrains early Cambrian geography and climate
Source: Nat Commun. 2021 Jun 23;12:3868. doi: 10.1038/s41467-021-24141-5 (PMC8222365; doi:10.1038/s41467-021-24141-5)
Supplement: Supplementary file 1 — Supplementary Information [file 41467_2021_24141_MOESM1_ESM.pdf]

Supplementary Information for: Quantitative comparison of geological data and model simulations constrains early Cambrian geography and climate

Thomas W. Wong Hearing[1,2], Alexandre Pohl[3,4], Mark Williams[2], Yannick Donnadieu[5], Thomas H. P. Harvey[2], Christopher Scotese[6], Pierre Sepulchre[7,8], Alain Franc[9], Thijs R. A. Vandenbroucke[1]

[1]Department of Geology, Ghent University, 9000, Belgium

[2]School of Geography, Geology and the Environment, University of Leicester, Leicester, LE1 7RH, UK

[3]Department of Earth and Planetary Sciences, University of California, Riverside, CA, USA

[4]Biogéosciences, UMR 6282, UBFC/CNRS, Université Bourgogne Franche-Comté, 6 boulevard Gabriel, F-21000 Dijon, France

[5]Aix-Marseille Univ, CNRS, IRD, INRA, Coll. France, CEREGE, Aix-en-Provence, France

[6]Department of Earth & Planetary Sciences, Northwestern University, Evanston, IL, USA

[7]Laboratoire des Sciences du Climat et de l'Environnement, LSCE/IPSL, CEA-CNRS-UVSQ, Université Paris-Saclay, F-91191 Gif-sur-Yvette, France

[8]INRAE, University of Bordeaux, BIOGECO, F-33610 Cestas, France

[9]Inria Bordeaux-Sud-Ouest, Pleiade, F-33405 Talence, France

Correspondence to: Thomas W. Wong Hearing: thomas.wonghearing@ugent.be

Alexandre Pohl: alexandre.pohl@u-bourgogne.fr

Mark Williams: mri@leicester.ac.uk

Contents

Supplementary Notes 1: Climatically sensitive lithologies 2

Supplementary Notes 2: Modelling Cambrian climate........ 9

Supplementary Notes 3: Köppen–Geiger climate classification 22

Supplementary Notes 4: Data–Model comparison 35

Supplementary References 43

Supplementary Notes 1: Climatically sensitive lithologies

Supplementary methods. The lithologies used in this study and their climatic conditions of formation are outlined in Table 1 in the main text. All climatically sensitive lithologies included in our analyses were identified as 'lower Cambrian' strata (approximately equivalent to the Terreneuvian Series and Cambrian Series 2). The lack of formal definition of Cambrian Series 2 makes it more challenging to confidently identify strata belonging to this interval. Given the challenges inherent to correlating these strata globally[e.g. 1–3], we consider series-level stratigraphic resolution appropriate for this study. We analysed two subsets of the lithology database, selecting in separate analyses either (i) traditional 'lower Cambrian' deposits, or (ii) deposits likely belonging to Cambrian Series 2.

Supplementary results. Cambrian Series 2 deposits are shown on each continental configuration in Supplementary Figure 1, the counterpart to main Figure 1. Lower Cambrian and Cambrian Series 2 deposits in the dataset are summarised by palaeocontinent in Supplementary Figure 2. The zonal distribution of lower Cambrian and Cambrian Series 2 strata on each continental configuration are summarised in 10° and 5° palaeolatitudinal bins in Supplementary Figure 3 to Supplementary Figure 6.

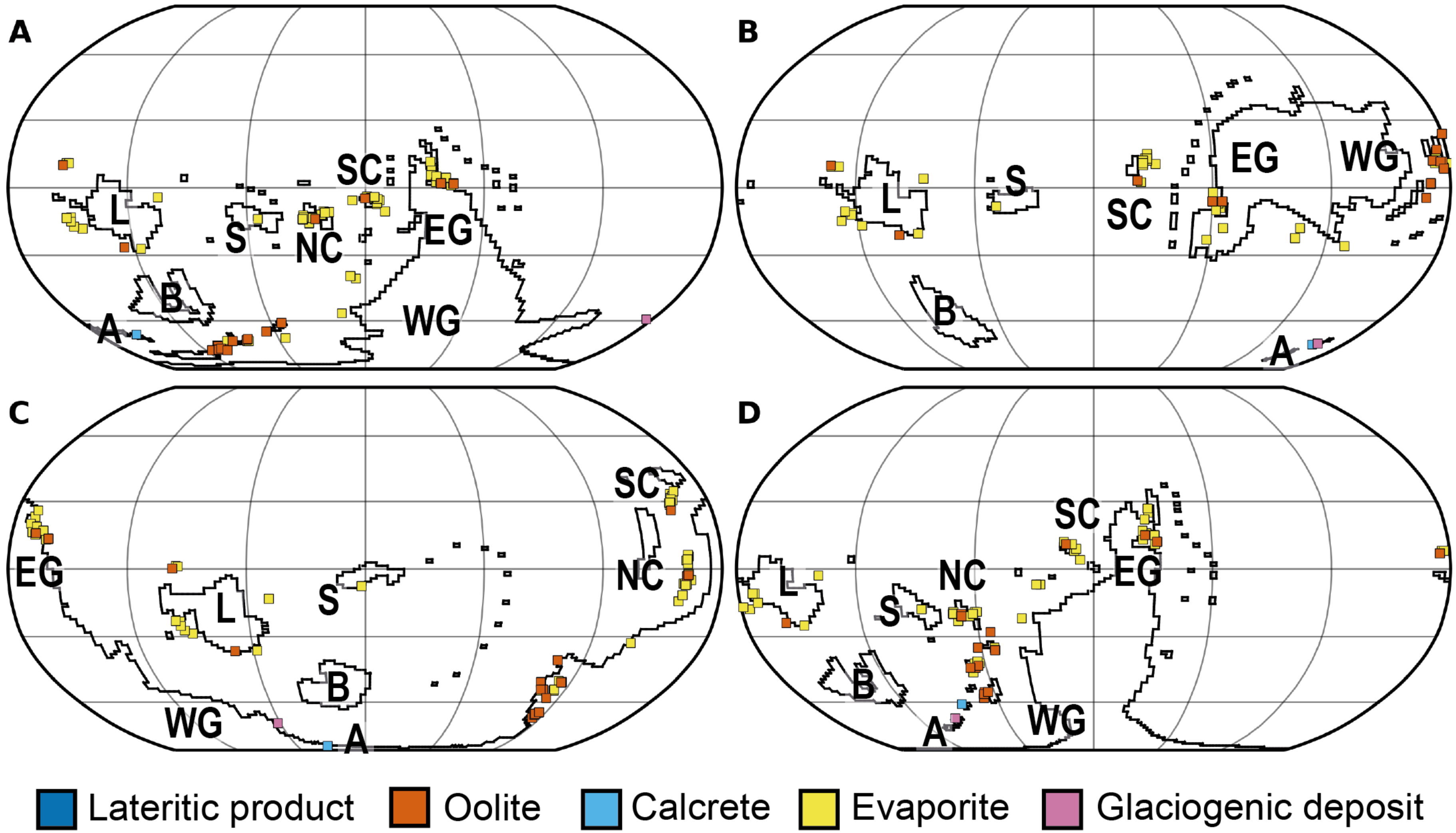

Supplementary Figure 1. Continental configurations (A–D) with the palaeo-positions of Cambrian Series 2 climatically sensitive lithologies (counterpart to main Figure 1). See Figure 1 for explanation of the continental configurations. Palaeocontinent abbreviations as follows: A: Avalonia; B: Baltica: EG: East Gondwana; L: Laurentia; NC: North China; S: Siberia; SC: South China; WG: West Gondwana.

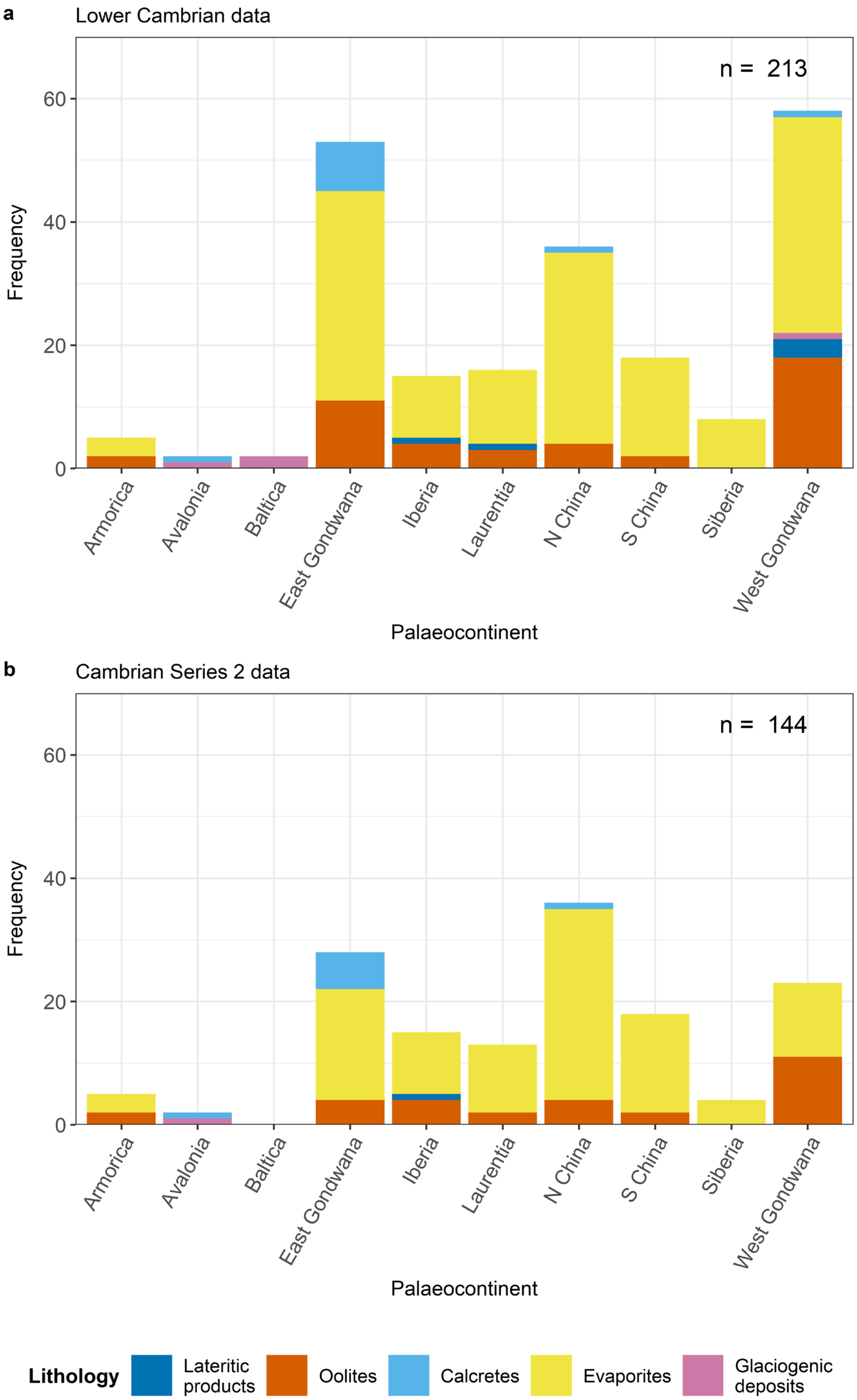

Supplementary Figure 2. Palaeocontinental distribution of lithology data. a Lower Cambrian data. **b** Cambrian Series 2 data.

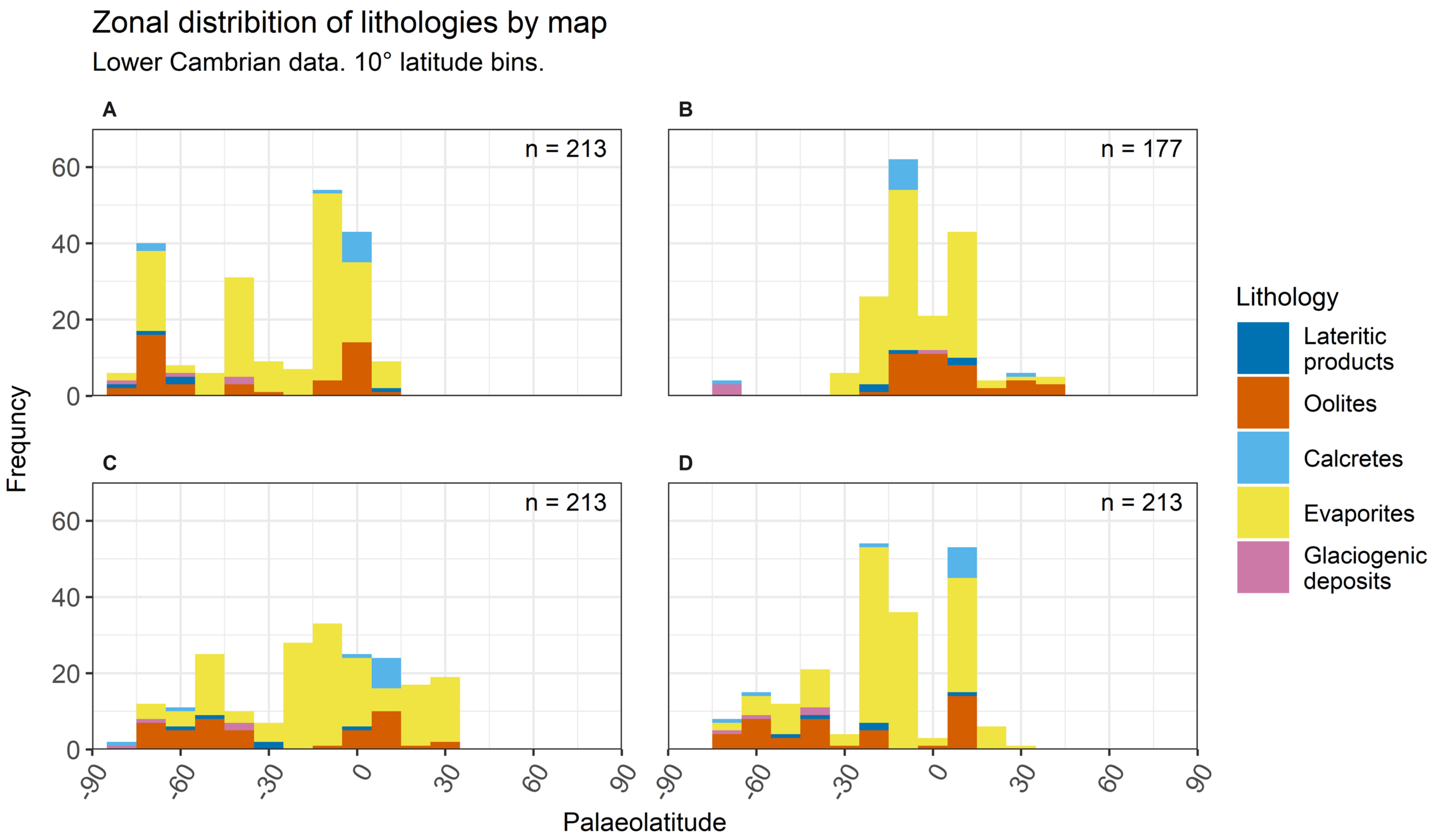

Supplementary Figure 3. Zonal distribution (10° bins) of lower Cambrian climatically sensitive lithologies on each continental configuration (A–D). This is a repeat of main text Figure 2 and is included here for ease of comparison in the supplementary files.

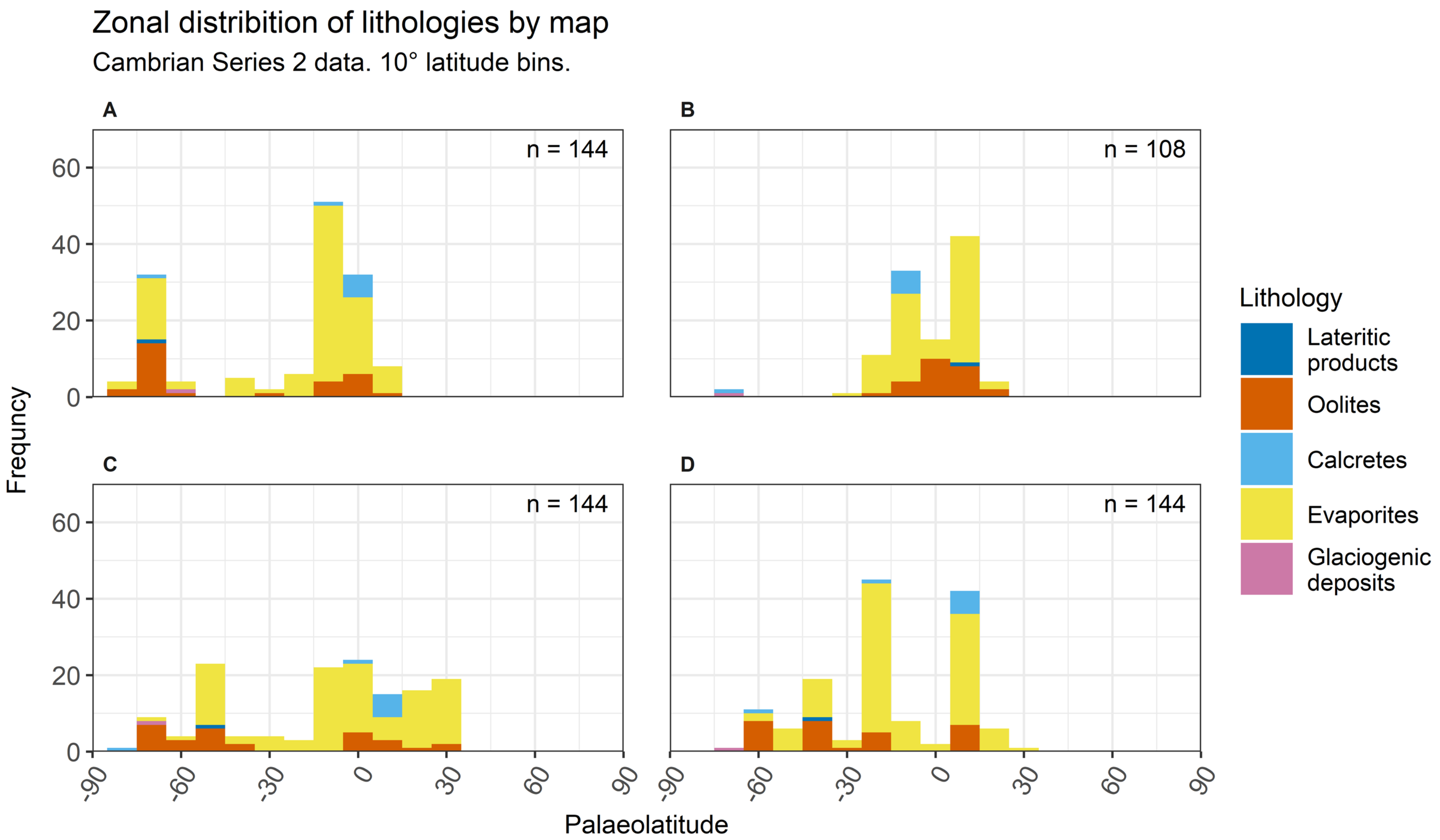

Supplementary Figure 4. Zonal distribution (10° bins) of Cambrian Series 2 climatically sensitive lithologies on each continental configuration (A–D).

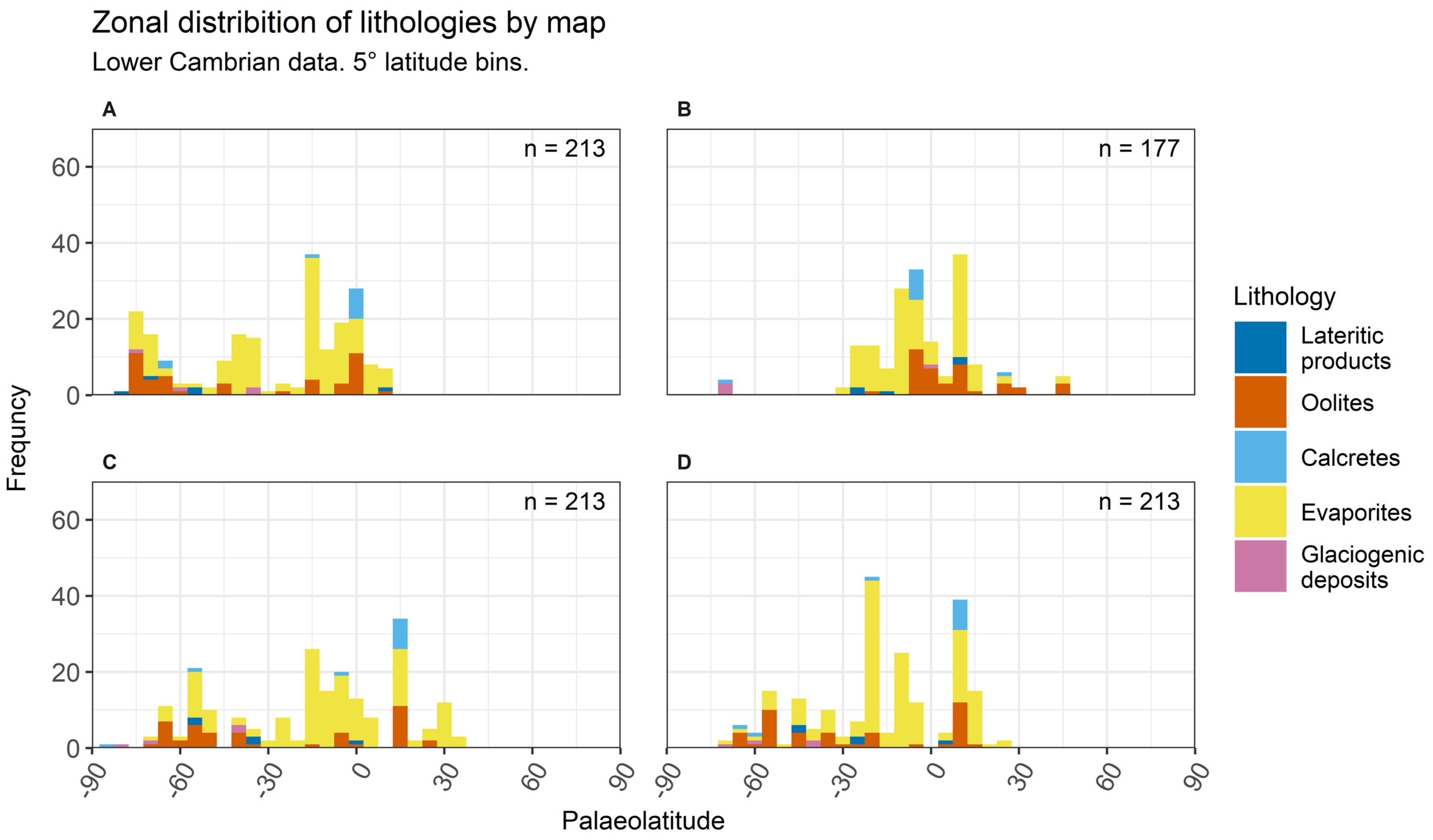

32

33 **Supplementary Figure 5. Zonal distribution (5° bins) of lower Cambrian climatically sensitive lithologies on each continental configuration (A–D).**

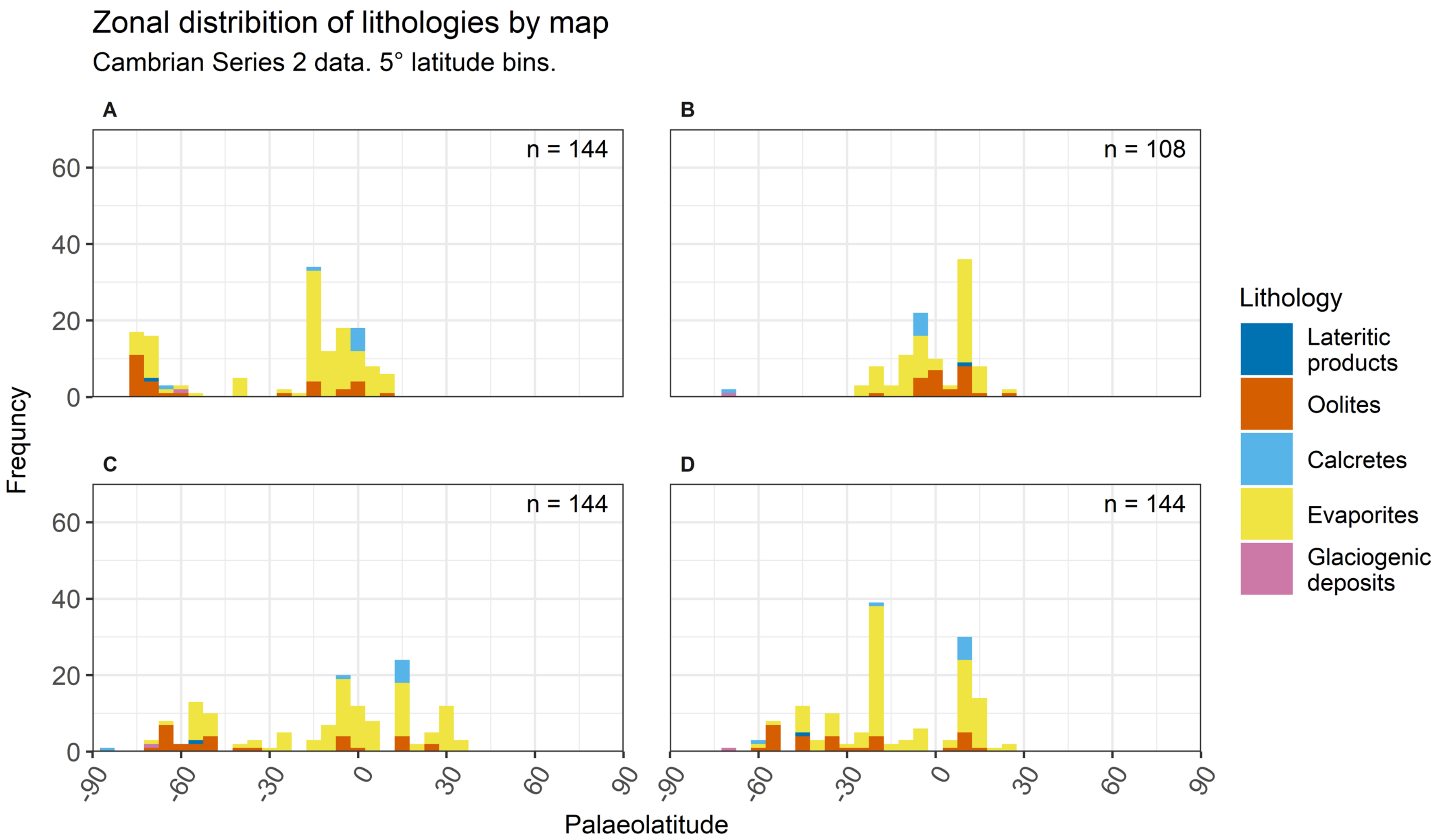

34

35 **Supplementary Figure 6. Zonal distribution (5° bins) of Cambrian Series 2 climatically sensitive lithologies on each continental configuration (A–D).**

Supplementary Notes 2: Modelling Cambrian climate

Supplementary methods. Estimates for atmospheric pCO_2 levels from long-term carbon cycle models are reported in Supplementary Table 1. The values used in this study are 4, 8 , 16, 32, 64, and 128 times preindustrial atmospheric level (PAL = 280 ppm pCO_2). The orbital parameters used in this study are reported in Supplementary Table 2. Supplementary Table 3 details the altitude/bathymetry categories used to produce the three-dimensional base maps.

Supplementary results. Modelled mean annual sea surface temperature (SST) values for each continental configuration under all tested orbital parameters and pCO_2 forcings are presented in Supplementary Figure 7. Mean annual SST maps for all simulations are presented in Supplementary Figure 8 to Supplementary Figure 17. Modelled zonal SST gradients for each continental configuration under all tested orbital conditions and pCO_2 forcings are summarised in Supplementary Figure 18. A summary of tropical and polar SSTs simulated with present day orbital parameters and 16 and 32 PAL pCO_2 values is given in Supplementary Table 4. Modelled sea ice fraction for each continental configuration under all tested orbital conditions and pCO_2 forcings is summarised in Supplementary Figure 19. Modelled mean annual surface air temperatures (MAATs) are presented in Supplementary Figure 20. Modelled mean annual precipitation (MAP) for each continental configuration under all tested orbital configurations and pCO_2 forcings is presented in Supplementary Figure 21. There is a highly non-linear response in sea and air temperatures, precipitation, and sea ice in continental configurations B and C between 8 PAL and 4 PAL pCO_2, a result that is comparable to that of ref. [4] who found pronounced climatic instability between 8 PAL and 6 PAL pCO_2 in their Ordovician climate simulations. The relationship between ocean area and modelled mean annual surface air temperature is shown in Supplementary Figure 22.

Supplementary Table 1. Geochemical-climate model predictions of early Cambrian atmospheric CO_2.

Model	Conditions	pCO_2 (PAL[a])	Reference
COPSE	Best estimate	~13	Ref. [5] fig. 13
	Sensitivity tests	~8 to ~18	Ref. [5] fig. 13
GEOCARBSULF	Median	~25	Ref. [6] figs. 2, 5
	All calculations (95 % CI)	~13 to ~96	Ref. [6] fig. 5
	75 % input reduction (95 % CI)	~16 to ~35	Ref. [6] fig. 5
GEOCARBSULFOR	Cambrian range	~22 to ~29	Ref. [7] supplementary fig. 5
GEOCLIM	Full 3D	>30	Ref. [8] fig. 8
	GEOCLIMtec limited forcing	~28	Ref. [8] fig. 8

[a]1 PAL = 280 ppm for all models.

Supplementary Table 2. Orbital parameters for the FOAM climate simulations.

Name	Description	Obliquity	Eccentricity	Longitude of perihelion
PD	Present day	23.4463	0.016724	77.9610
HS	Hot austral summer	24.5	0.07	90 (perihelion December)
CS	Cold austral summer	24.5	0.07	270 (perihelion June)
OL	Obliquity minimum	22.0	0.0	90
OH	Obliquity maximum	24.5	0.0	90

Supplementary Table 3. Altitude categories used for palaeogeographic reconstructions, following ref. [4].

Category	Altitude (m)	Evidence
Active orogeny	4000	High pressure metamorphic rocks dated 515 Ma ± 20 Ma. Inferred to be linear along continental or craton boundaries.
Old orogeny	2000	High pressure metamorphic rocks dated 535 Ma to 600 Ma. Inferred to be linear along continental or craton boundaries.
Continental plain	200	Terrestrial rocks not indicative of active or old orogenies. Depositional hiatus adjacent to shallow marine rocks.
Marine shallow shelf	−100	Shallow marine rocks inferred to have been deposited above storm wave base. Stromatolites are also considered evidence of shallow marine deposition.
Marine deep shelf	−200	Marine rocks inferred to have been deposited below storm wave base, including turbidite deposits, hemipelagic mudstones. Distal margin determined by boundaries of continental blocks.
Abyssal ocean	−4000	Offshore of deep shelf settings.

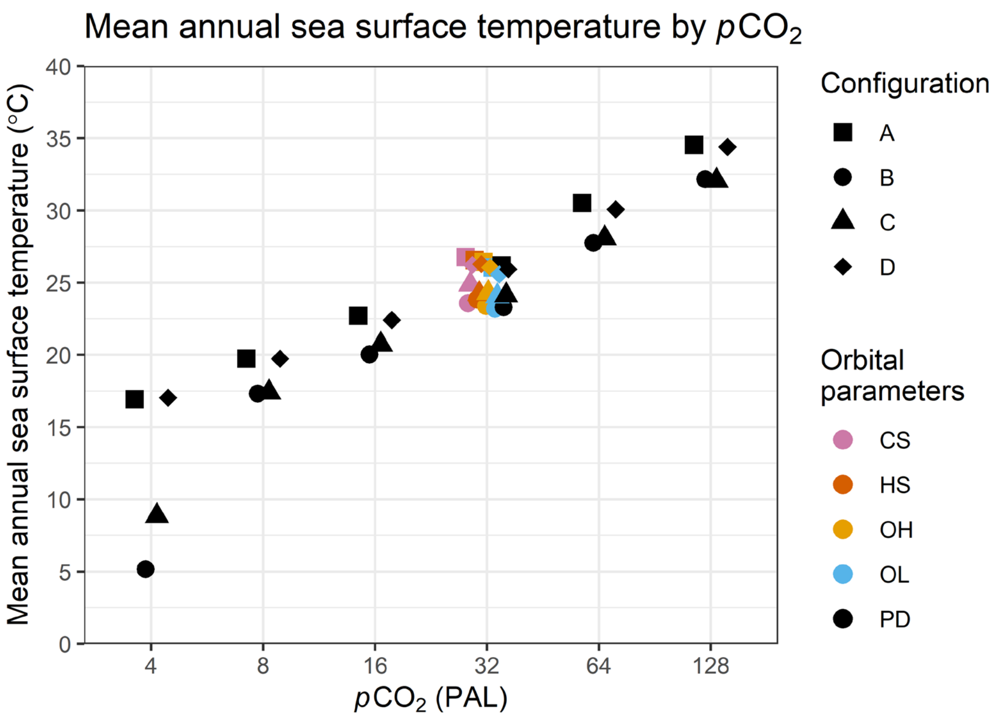

Supplementary Figure 7. Mean annual SSTs from each simulation by greenhouse gas forcing, orbital parameters, and continental configuration. See Supplementary Table 2 for explanation of the orbital parameters.

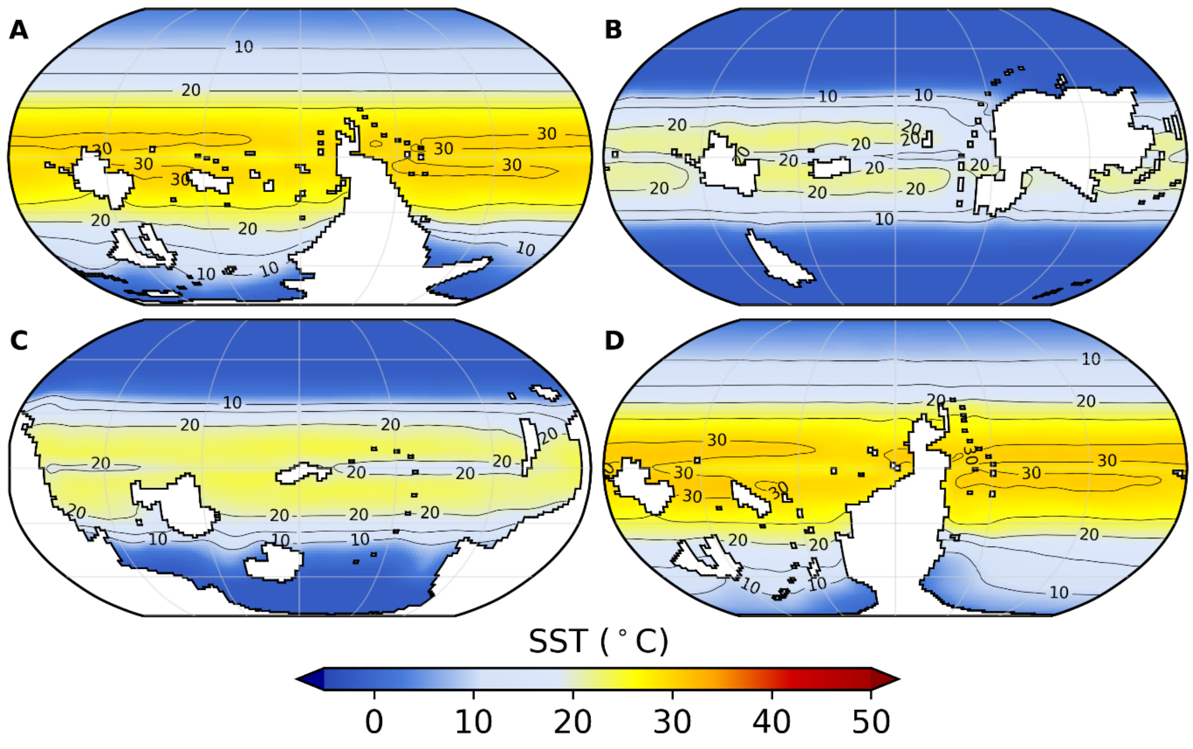

Supplementary Figure 8. Mean annual SSTs for configurations A–D for a present day orbit at 4 PAL CO_2.

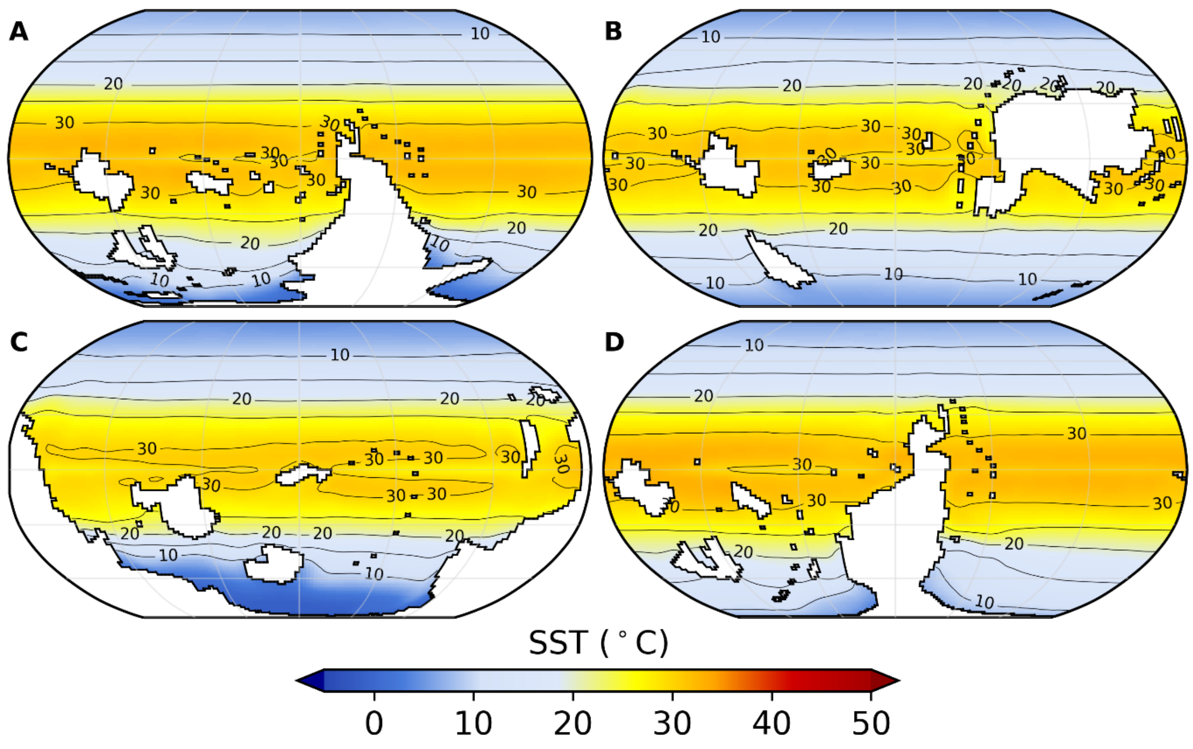

Supplementary Figure 9. Mean annual SSTs for configurations A–D for a present day orbit at 8 PAL CO_2.

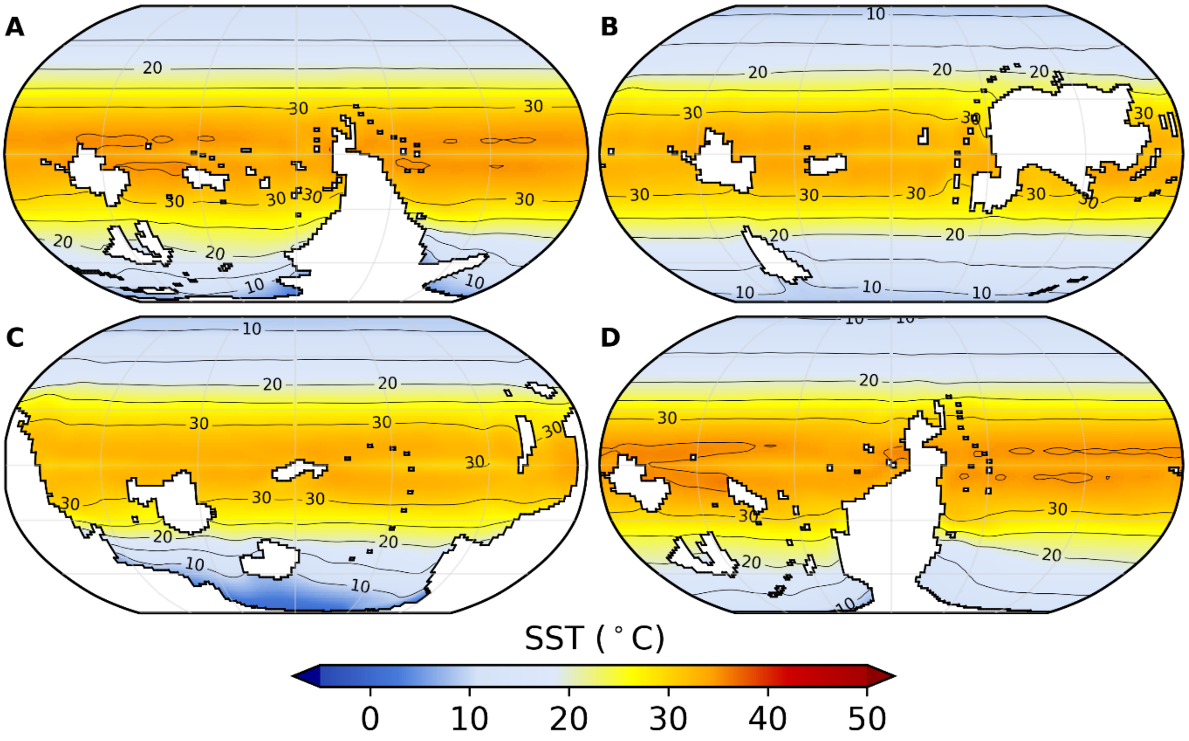

Supplementary Figure 10. Mean annual SSTs for configurations A–D for a present day orbit at 16 PAL CO_2.

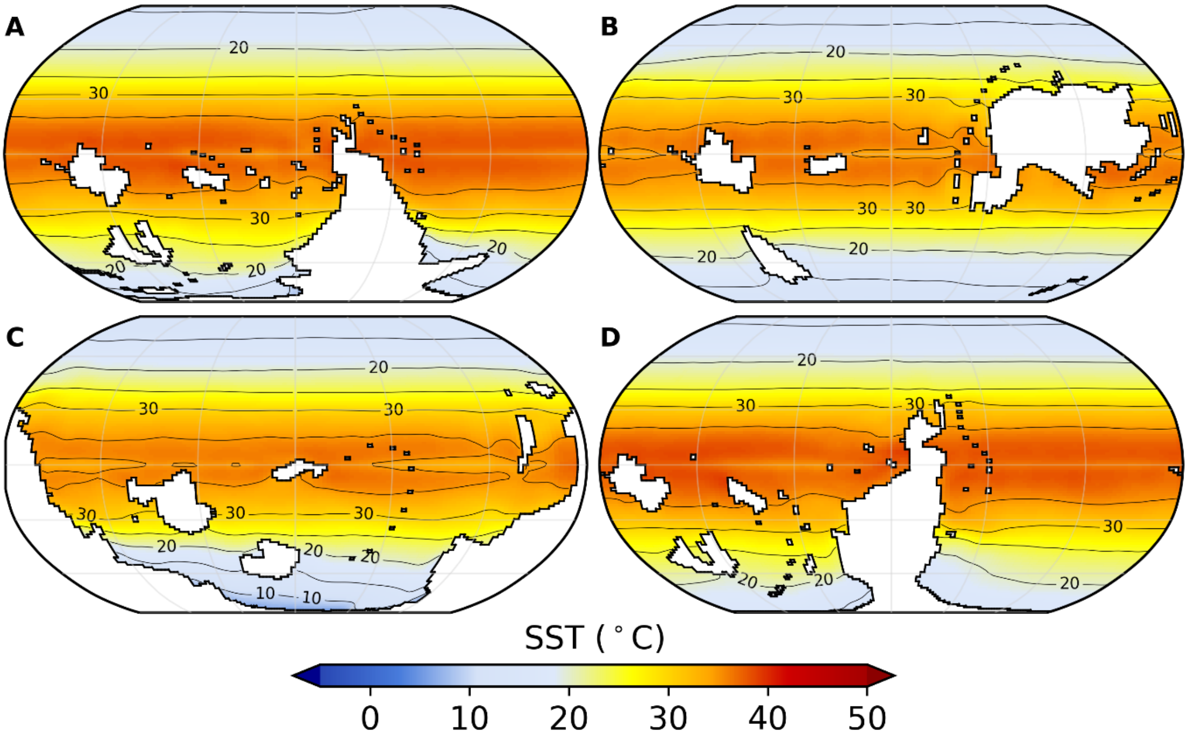

Supplementary Figure 11. Mean annual SSTs for configurations A–D for a present day orbit at 32 PAL CO_2.

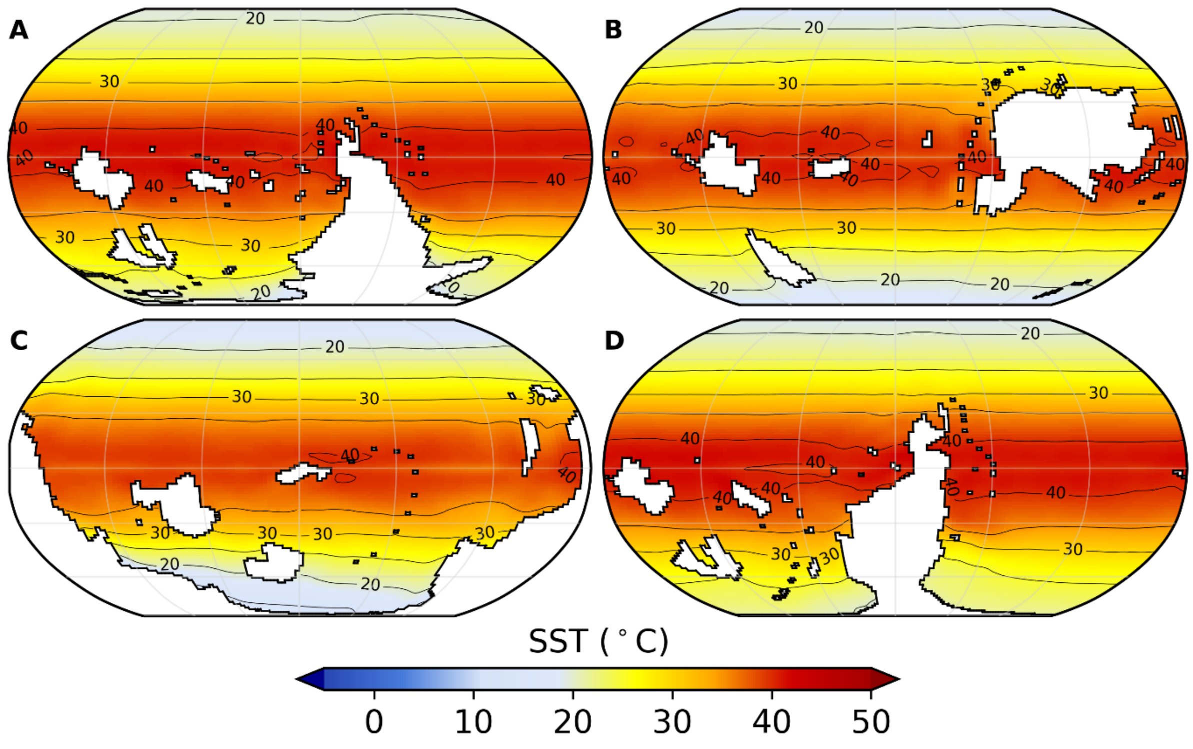

Supplementary Figure 12. Mean annual SSTs for configurations A–D for a present day orbit at 64 PAL CO_2.

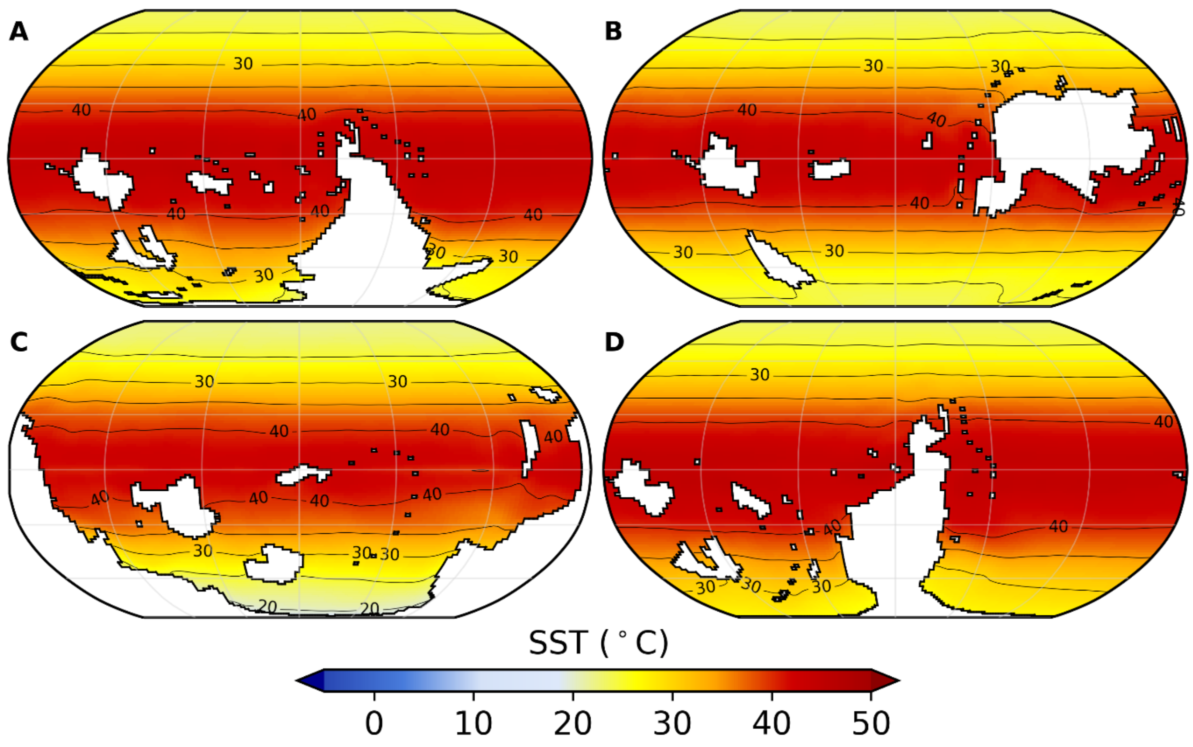

Supplementary Figure 13. Mean annual SSTs for configurations A–D for a present day orbit at 128 PAL CO_2.

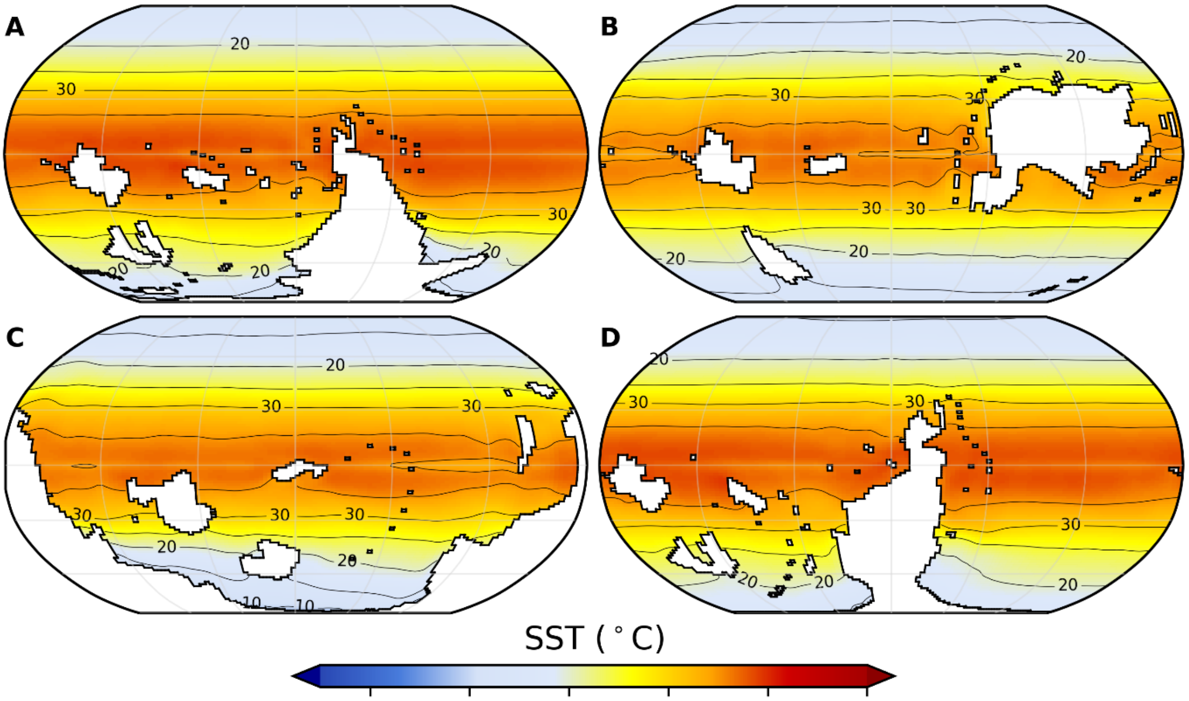

Supplementary Figure 14. Mean annual SSTs for configurations A–D for a cold austral summer orbit at 32 PAL CO_2.

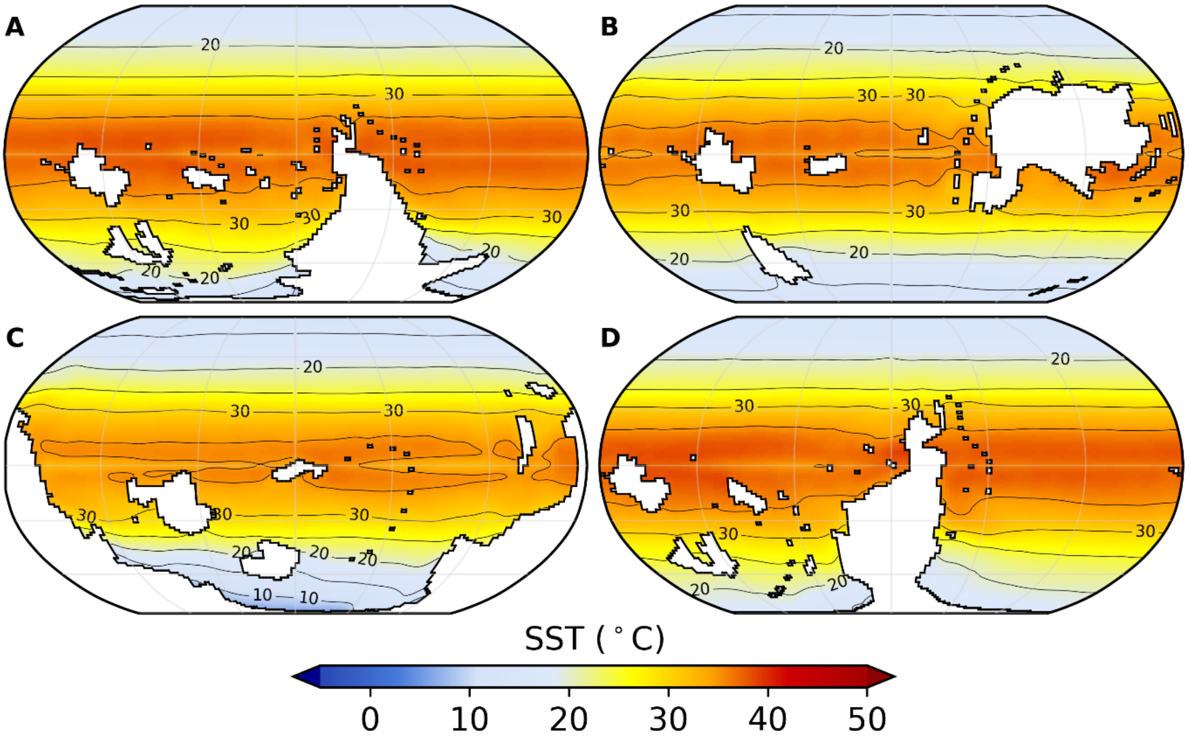

Supplementary Figure 15. Mean annual SSTs for configurations A–D for a hot austral summer orbit at 32 PAL CO_2.

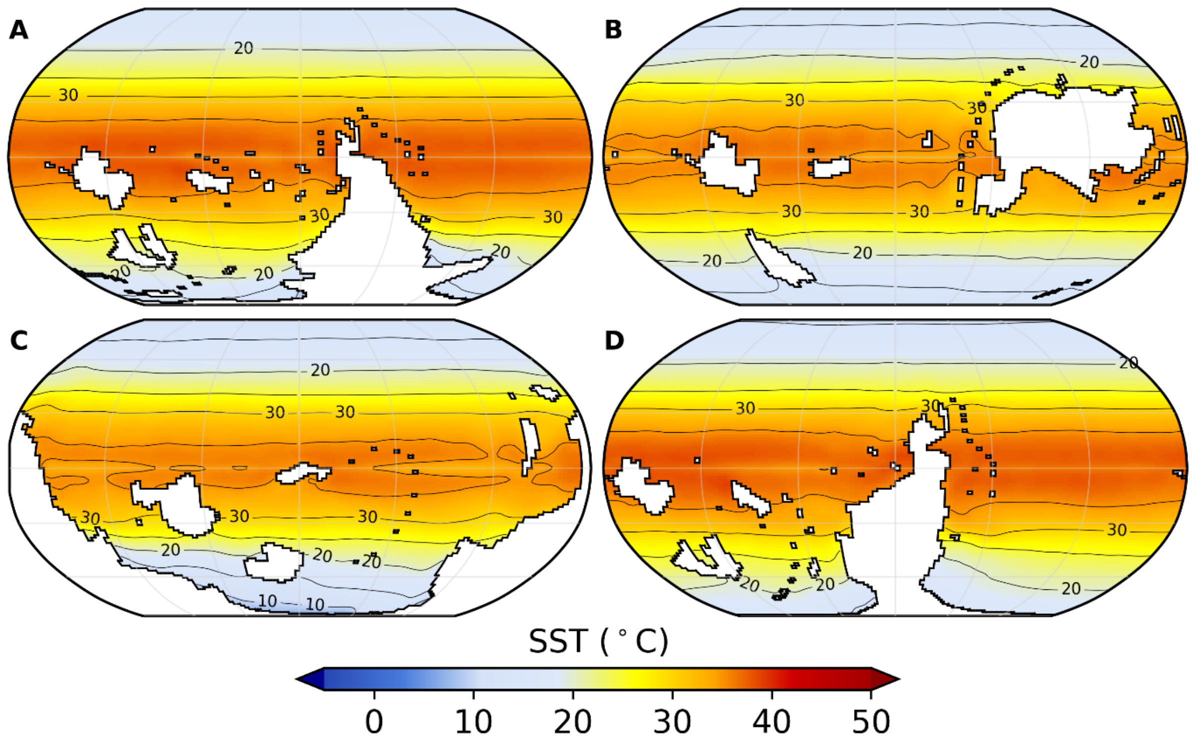

Supplementary Figure 16. Mean annual SSTs for configurations A–D for a high obliquity orbit at 32 PAL CO_2.

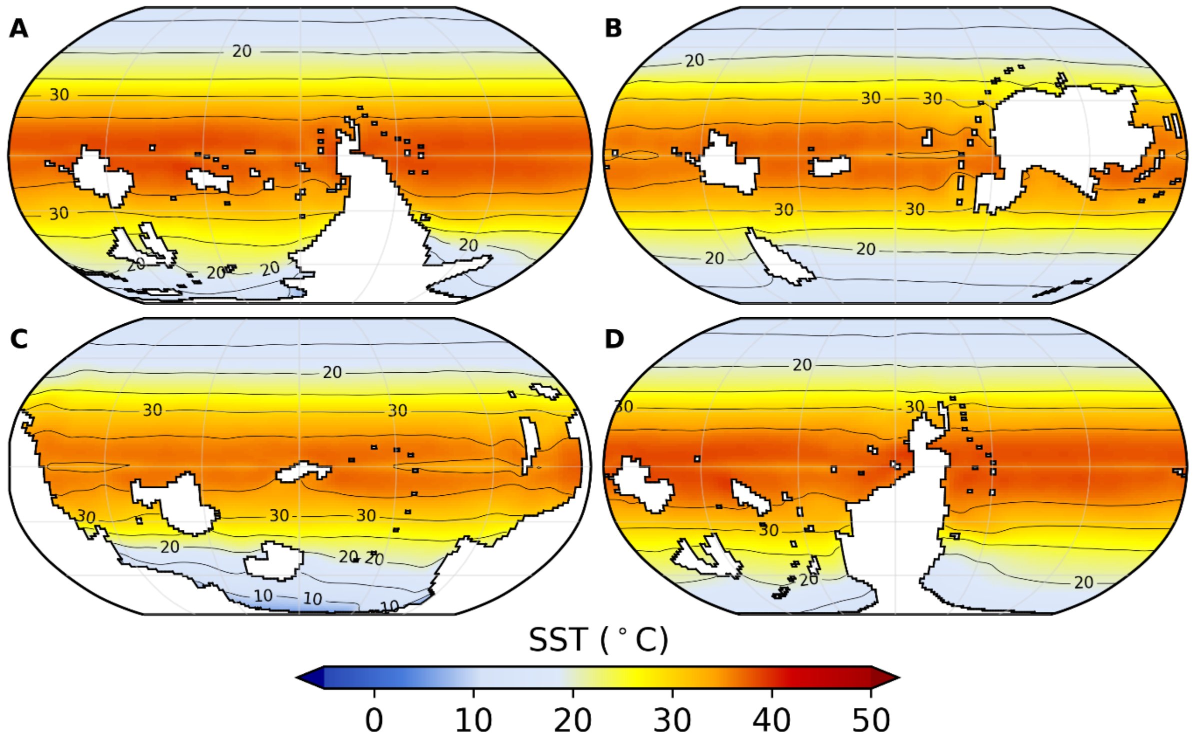

Supplementary Figure 17. Mean annual SSTs for configurations A–D for a low obliquity orbit at 32 PAL CO_2.

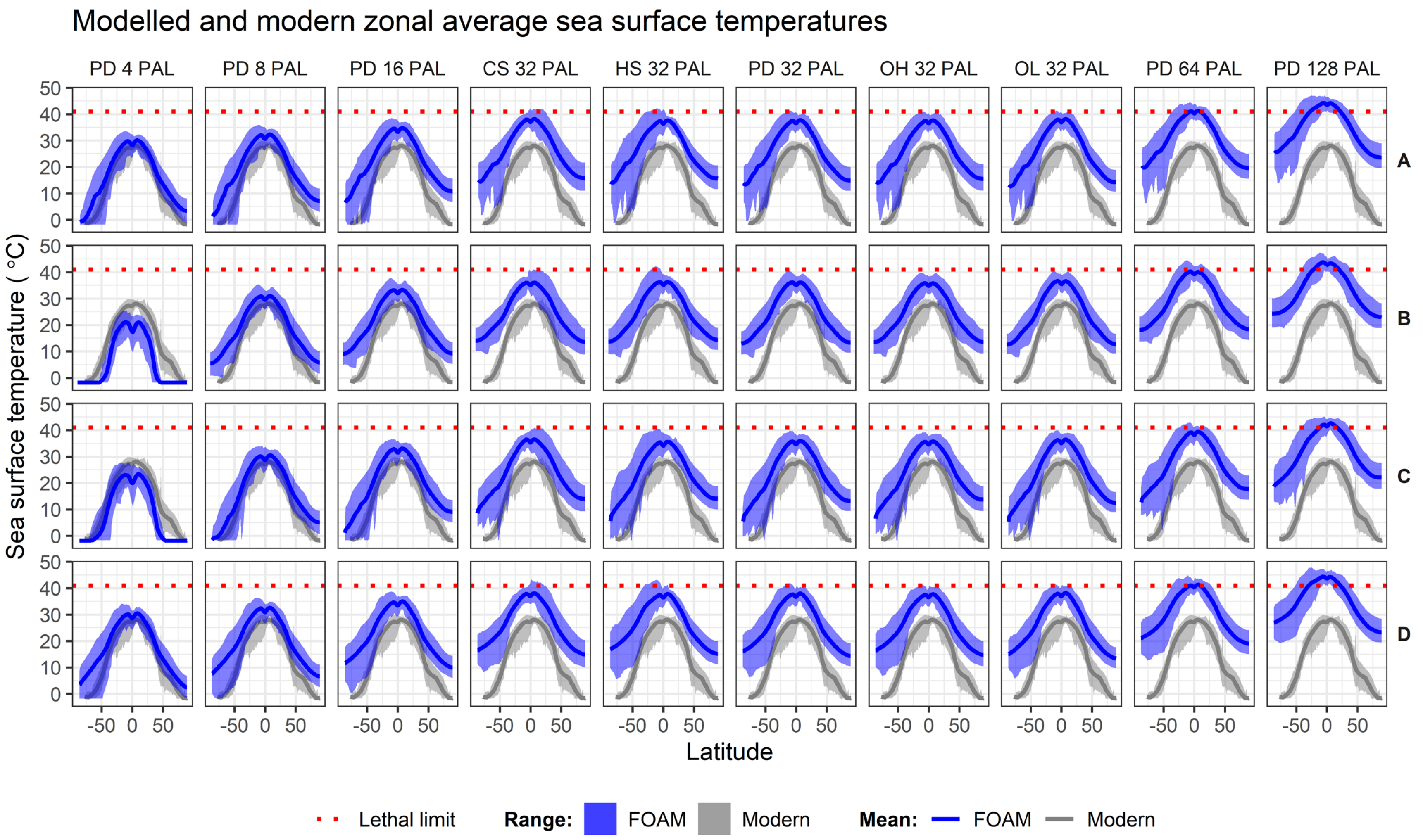

Supplementary Figure 18. Zonally averaged model (blue) sea surface temperature (SST) annual mean and seasonal variation for each GCM simulation, with comparable present day SST values (grey)[9] and lethal temperature limit for marine animals (horizontal red line) of 41 °C following ref. [10]. See Supplementary Table 2 for explanation of the orbital parameters. $p$$CO_2$ levels relative to PAL (= 280 ppm).

Supplementary Table 4. Summary of tropical and polar sea surface temperatures in each FOAM simulation with present day (PD) orbital parameters, and 16 and 32 PAL pCO_2 values.

		Sea surface temperature (°C)							
		Configuration A		Configuration B		Configuration C		Configuration D	
		PD 16	PD 32	PD 16	PD 32	PD 16	PD 32	PD 16	PD 32
Tropical[a]	Min	30.6	33.6	29.5	32.3	28.7	31.5	30.6	33.6
	Mean	33.3	36.3	32.1	35.0	31.7	34.6	33.5	36.5
	Max	34.8	37.8	33.3	36.2	33.1	36.0	35.0	38.1
Polar[b]	Min	6.5	12.8	9.1	13.0	1.2	5.5	10.0	14.4
	Mean	11.0	15.5	10.6	14.4	7.8	12.3	12.3	16.5
	Max	13.9	18.0	13.3	16.7	12.2	15.9	15.4	19.2

[a]Tropical = latitude < ±24°.

[b]Polar = latitude ≥ ±66°.

Configurations A and C are shaded out to highlight continental configurations B and D which are supported by our analyses.

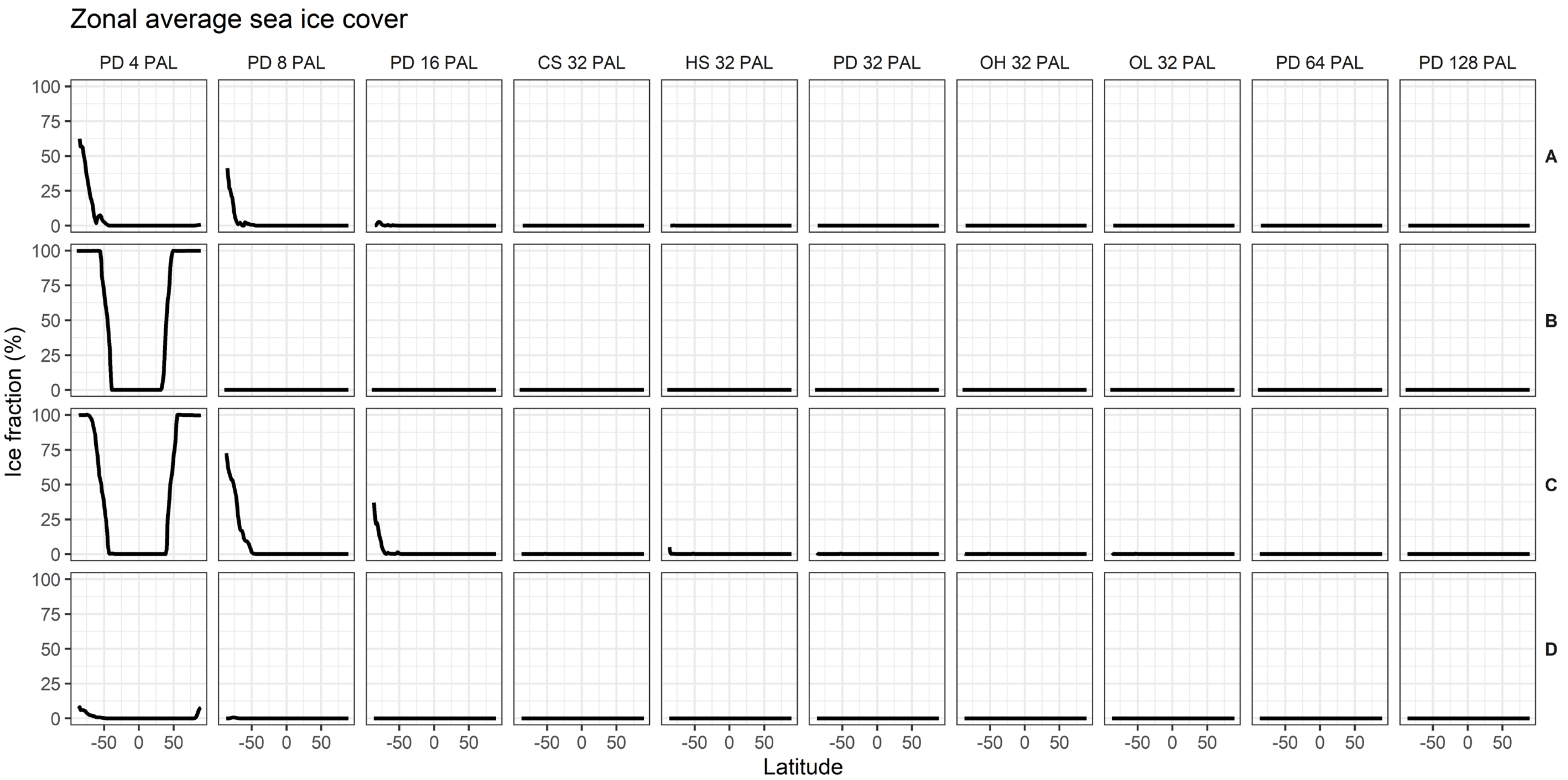

Supplementary Figure 19. Zonally averaged mean annual sea ice cover for each GCM simulation. See Supplementary Table 2 for explanation of the orbital parameters. $p\mathrm{CO_2}$ levels relative to PAL (= 280 ppm).

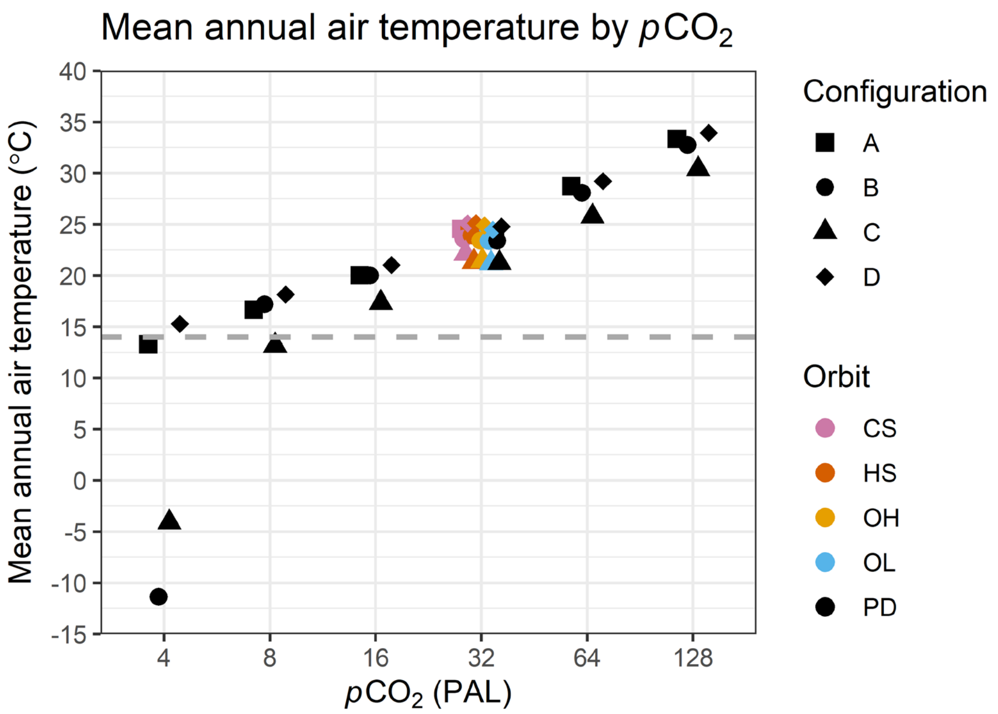

Supplementary Figure 20. Globally averaged mean annual surface air temperature (MAAT) for each GCM simulation. Dashed horizontal line = 14 °C modern MAAT[11]. See Supplementary Table 2 for explanation of the orbital parameters.

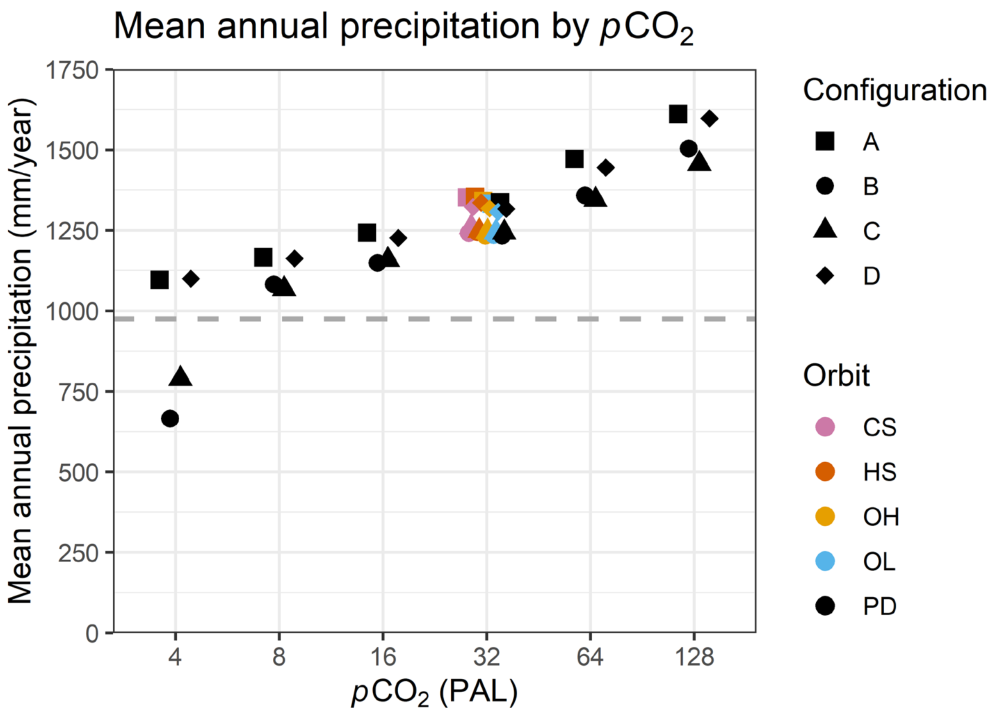

Supplementary Figure 21. Globally averaged mean annual precipitation (MAP) for each simulation. Dashed horizontal line represents present day global MAP = 975 mm/year (1979–2010 average)[12]. See Supplementary Table 2 for explanation of the orbital parameters.

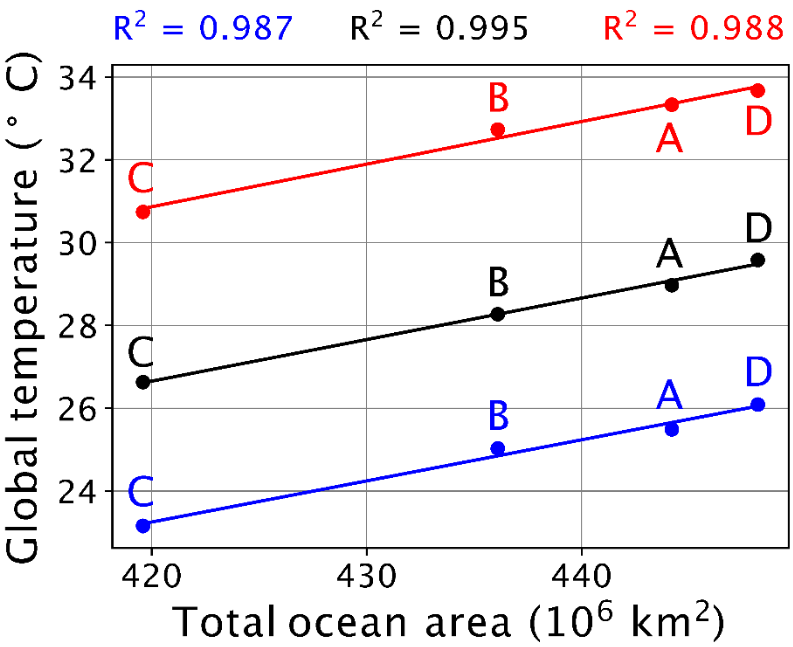

Supplementary Figure 22. Mean annual globally averaged modelled surface air temperatures at 16 PAL (blue), 32 PAL (black) and 64 PAL (red) as a function of the total ocean area for continental configurations A to D. Each point is a simulation conducted under present day orbital parameters. Lines represent linear regressions conducted for each pCO_2 level, the correlation coefficient of which is provided at the top of the figure using the same color code.

Supplementary Notes 3: Köppen–Geiger climate classification

Supplementary methods. The temperature and precipitation thresholds of the Köppen–Geiger climate classification are detailed in Supplementary Table 5. The Köppen–Geiger climate classes to which the lithology data were ascribed are detailed in Supplementary Table 6. See Table 1 for references to lithology formation conditions.

Supplementary results. Köppen–Geiger climate classes were calculated for each FOAM simulation in R (see Supplementary Data 3). The results of this script for each FOAM simulation are presented here as maps (Supplementary Figure 23 to Supplementary Figure 32). Köppen–Geiger class zonal relative frequency plots are included for interest (Supplementary Figure 33 to Supplementary Figure 42), but the reader is reminded that the regional rather than zonal patterns are most important in this study.

Supplementary Table 5. Primary and secondary class thresholds of the Köppen-Geiger climate classification, after ref. [13].

Primary climate	Secondary climate	Description	Criteria
A		Tropical	$T_{cold} \geq 18$
	f	fully humid	$P_{dry} \geq 60$
	m	monsoon	$P_{dry} < 60$ & $P_{dry} \geq 100–(MAP/25)$
	w	savannah	$P_{dry} < 60$ & $P_{dry} < 100–(MAP/25)$
B		Arid	$MAP < 10*P_{thresh}$
	W	Desert	$MAP < 5*P_{thresh}$
	S	steppe	$MAP \geq 5*P_{thresh}$
C		Temperate	$T_{hot} \geq 10$ & $0 < T_{cold} < 18$
	s	dry summer	$P_{sdry} < 40$ & $P_{sdry} < P_{wwet}/3$
	w	dry winter	$P_{wdry} < P_{swet}/10$
	f	no dry season	Not Cs or Cw
D		Cold	$T_{hot} \geq 10$ & $T_{cold} \leq 0$
	s	dry summer	$P_{sdry} < 40$ & $P_{sdry} < P_{wwet}/3$
	w	dry winter	$P_{wdry} < P_{swet}/10$
	f	no dry season	Not Ds or Dw
E		Polar	$T_{hot} < 10$
	T	tundra	$T_{hot} > 0$
	F	frost	$T_{hot} \leq 0$

T in °C; P in mm per month; MAP in mm per year.
Summer/Winter are defined as the warmer/cooler six-month period October to March (austral summer) or April to September (boreal summer).
MAP = mean annual precipitation; MAT = mean annual temperature.
P_{dry} = precipitation of driest month; P_{wet} = precipitation of wettest month; P_{sdry} = precipitation of driest summer month; P_{swet} = precipitation of wettest summer month; P_{wwet} = precipitation of wettest winter month.
T_{cold} = temperature of the coldest month; T_{hot} = temperature of hottest month.
If 70 % MAP in winter, P_{thresh} = 2 x MAT; if 70 % MAP in summer, P_{thresh} = (2 x MAT) + 28; else, P_{thresh} = (2 x MAT) + 14. P_{thresh} is therefore temperature-dependent.

Supplementary Table 6. Köppen-Geiger classes for each category of climactically sensitive lithology.

Lithology[a]		Köppen–Geiger class[b]												
Type	ID	Af	Am	Aw	BS	BW	Cs	Cw	Cf	Ds	Dw	Df	ET	EF
Calcretes	C			X	X	X	X	X						
Evaporites	E			X	X	X	X	X		X	X		X	
Glaciogenic deposits	G									X	X	X	X	X
Lateritic products	L	X	X	X										
Oolitic limestone	O	X	X	X	X	X								

[a]See Table 1 for definitions.

[b]See Supplementary Table 5 for Köppen–Geiger class definitions.

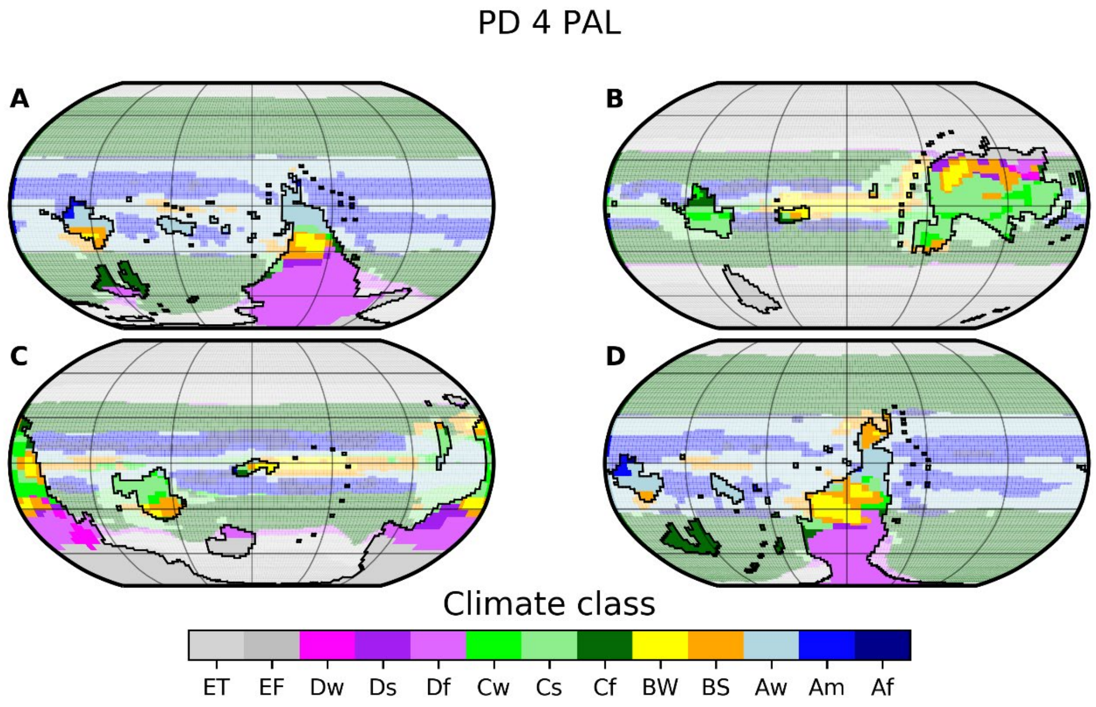

Supplementary Figure 23. Modelled Köppen–Geiger climate classes for a present day orbit at 4 PAL CO_2. See Supplementary Table 5 for climate classes and Supplementary Table 2 for orbital parameters.

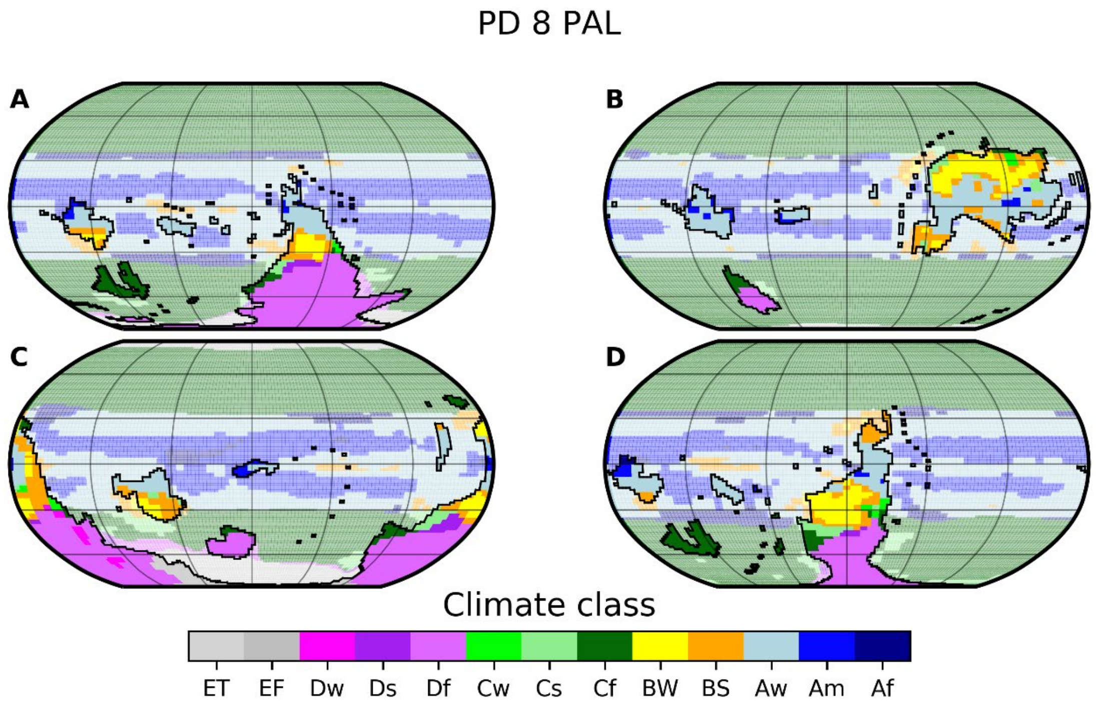

Supplementary Figure 24. Modelled Köppen–Geiger climate classes for a present day orbit at 8 PAL CO_2. See Supplementary Table 5 for climate classes and Supplementary Table 2 for orbital parameters.

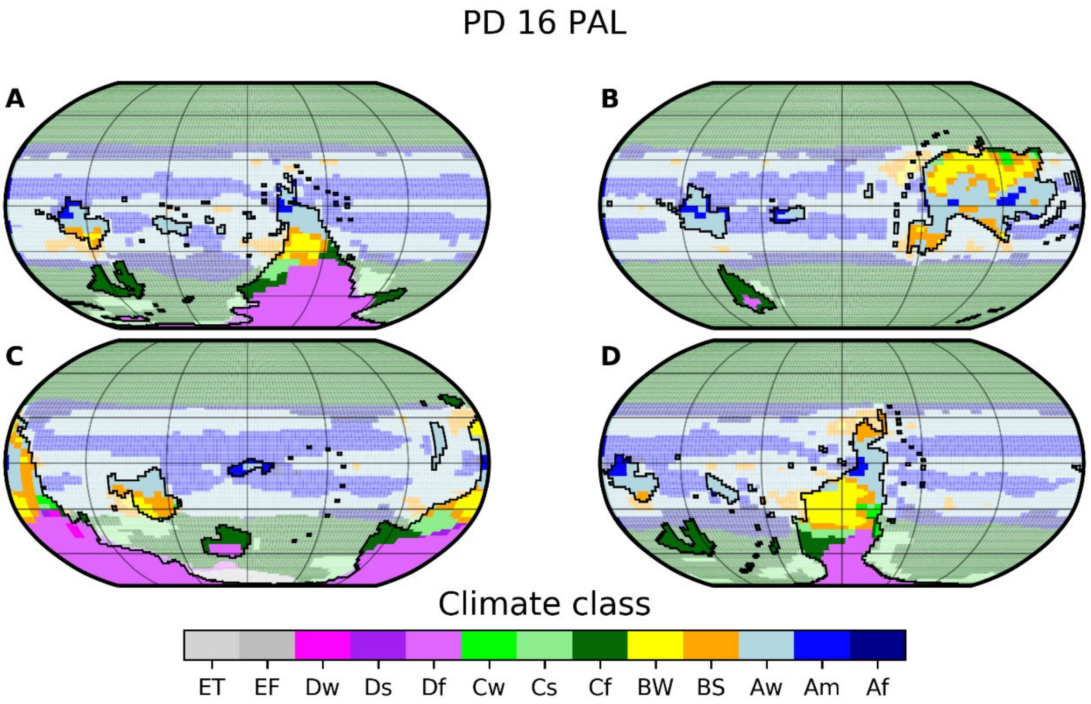

Supplementary Figure 25. Modelled Köppen–Geiger climate classes for a present day orbit at 16 PAL CO_2. See Supplementary Table 5 for climate classes and Supplementary Table 2 for orbital parameters.

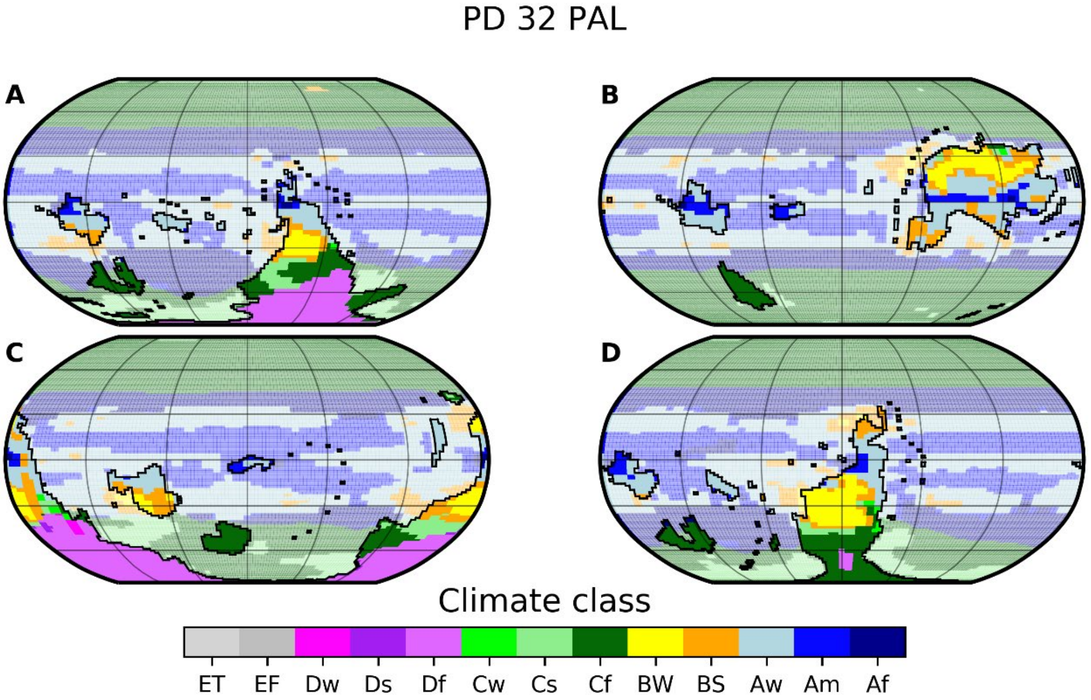

Supplementary Figure 26. Modelled Köppen–Geiger climate classes for a present day orbit at 32 PAL CO_2. See Supplementary Table 5 for climate classes and Supplementary Table 2 for orbital parameters.

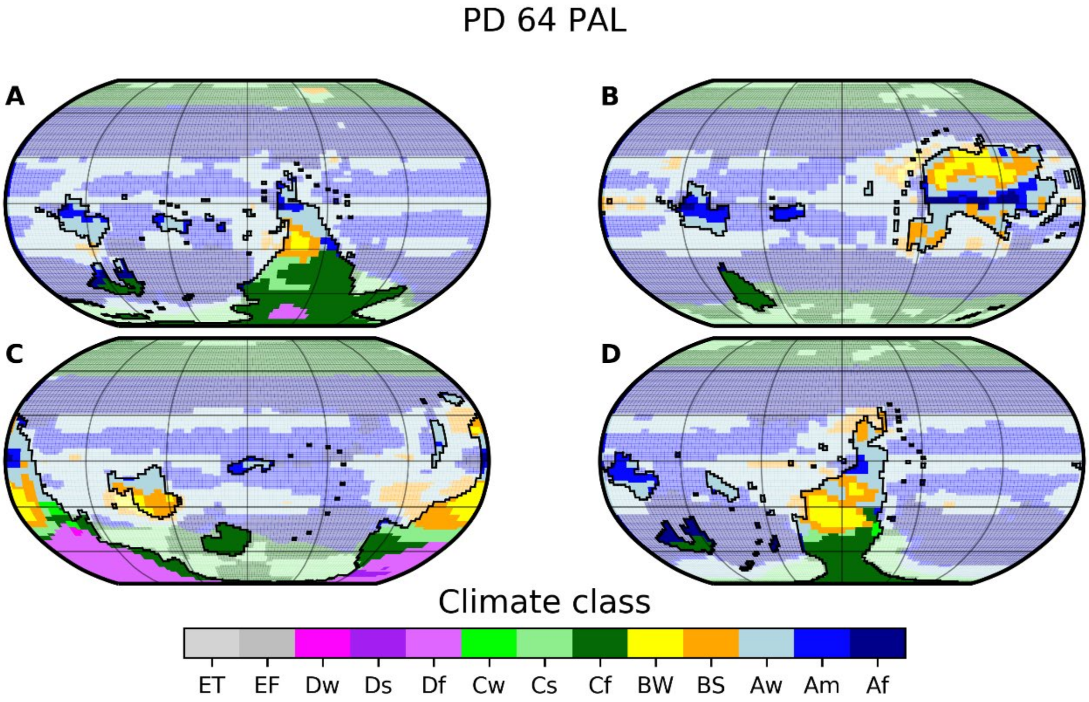

Supplementary Figure 27. Modelled Köppen–Geiger climate classes for a present day orbit at 64 PAL CO_2. See Supplementary Table 5 for climate classes and Supplementary Table 2 for orbital parameters.

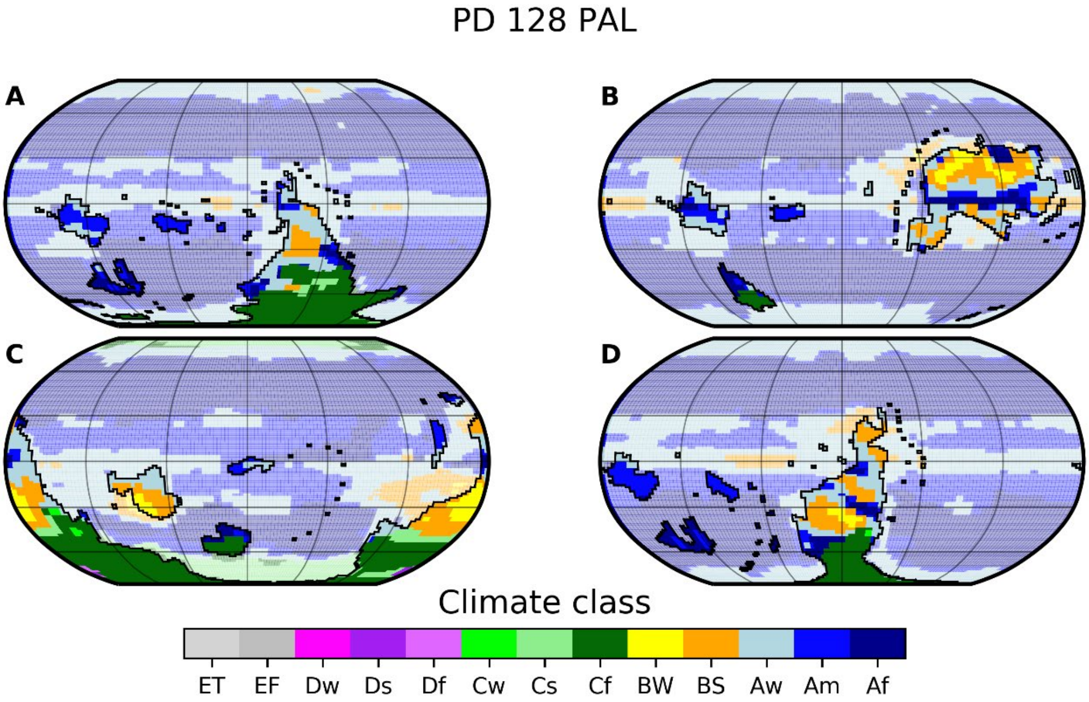

Supplementary Figure 28. Modelled Köppen–Geiger climate classes for a present day orbit at 128 PAL CO_2. See Supplementary Table 5 for climate classes and Supplementary Table 2 for orbital parameters.

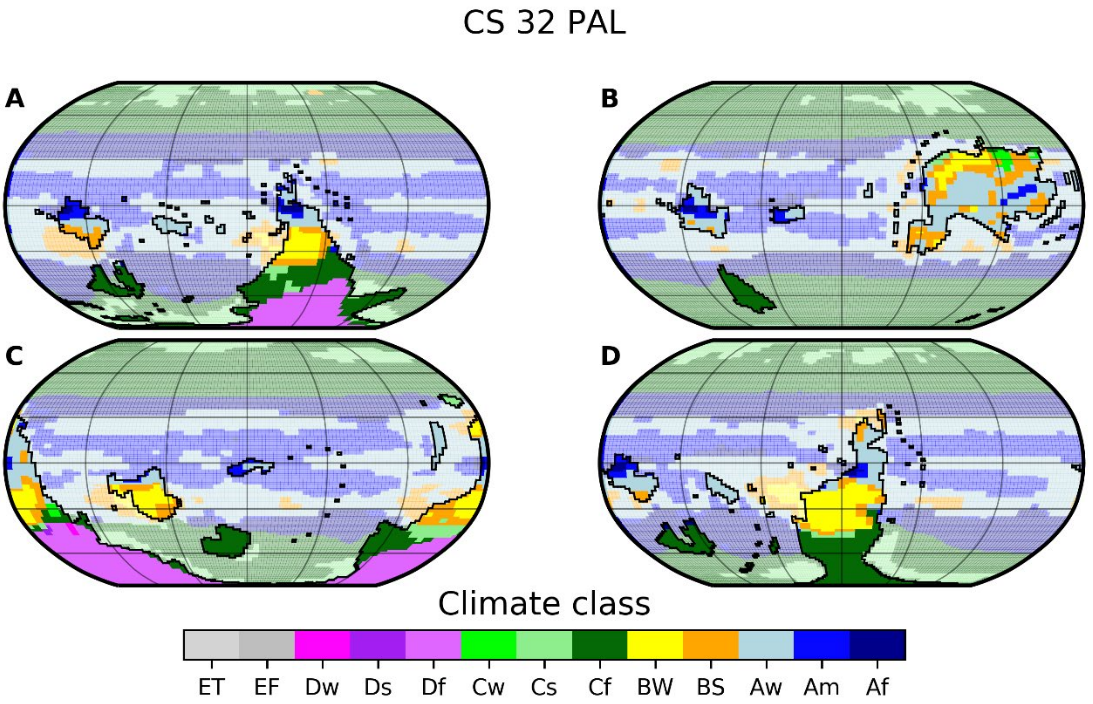

Supplementary Figure 29. Modelled Köppen–Geiger climate classes for a cold (austral) summer orbit at 32 PAL CO_2. See Supplementary Table 5 for climate classes and Supplementary Table 2 for orbital parameters.

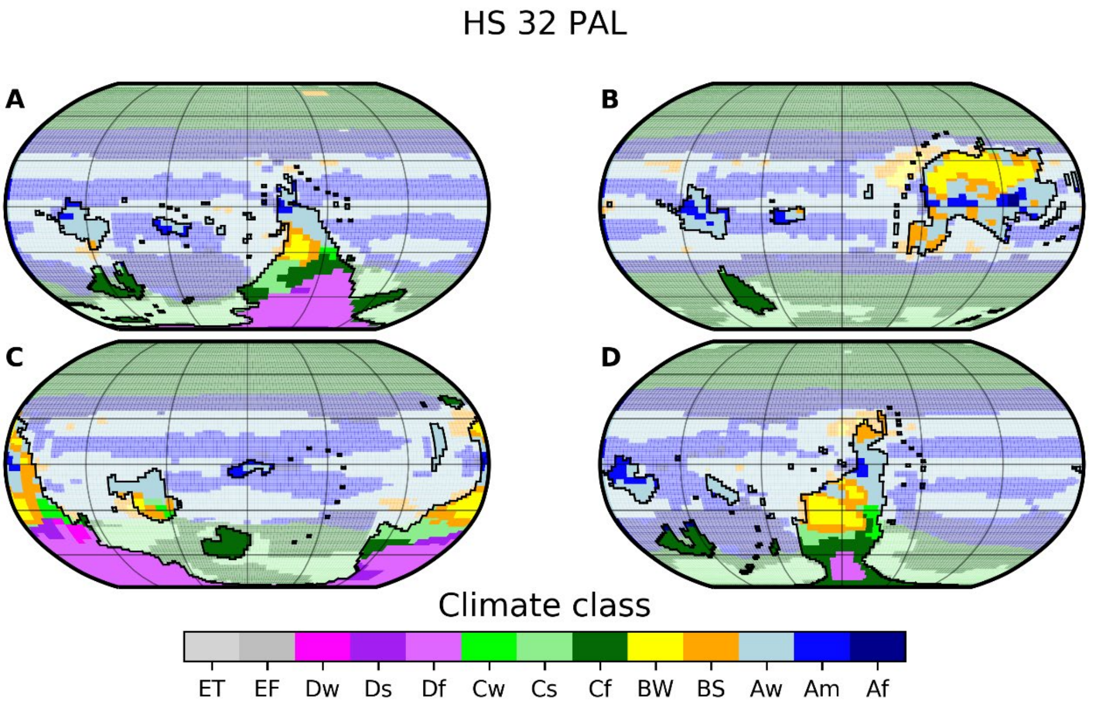

Supplementary Figure 30. Modelled Köppen–Geiger climate classes for a hot (austral) summer orbit at 32 PAL CO_2. See Supplementary Table 5 for climate classes and Supplementary Table 2 for orbital parameters.

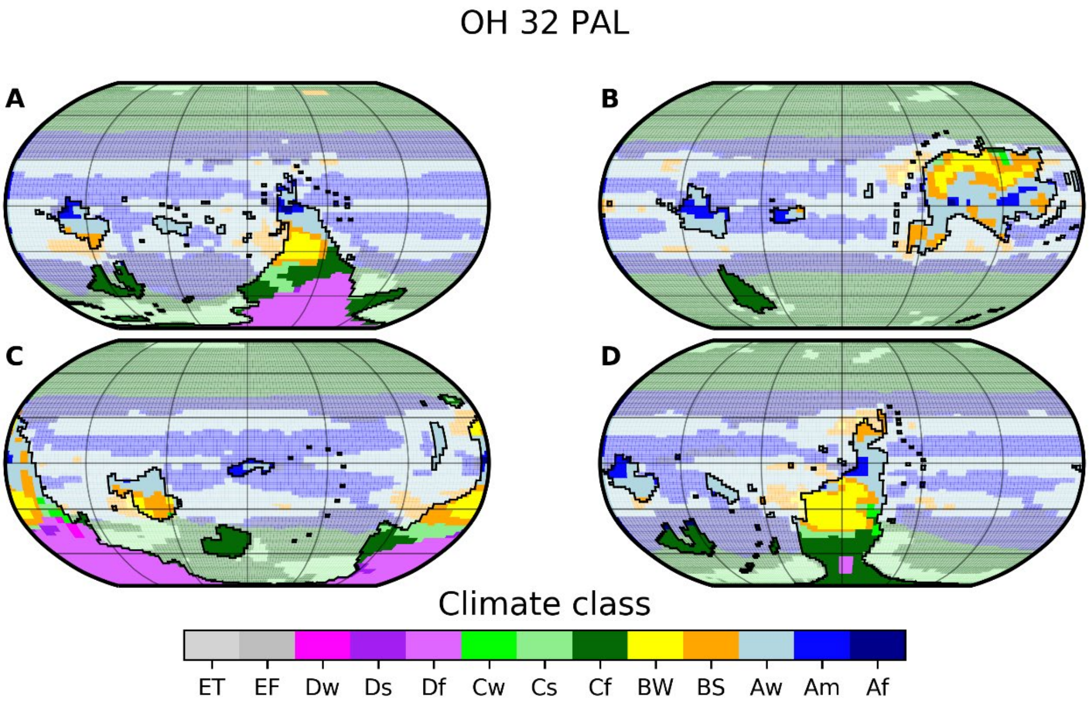

Supplementary Figure 31. Modelled Köppen–Geiger climate classes for a high obliquity orbit at 32 PAL CO_2. See Supplementary Table 5 for climate classes and Supplementary Table 2 for orbital parameters.

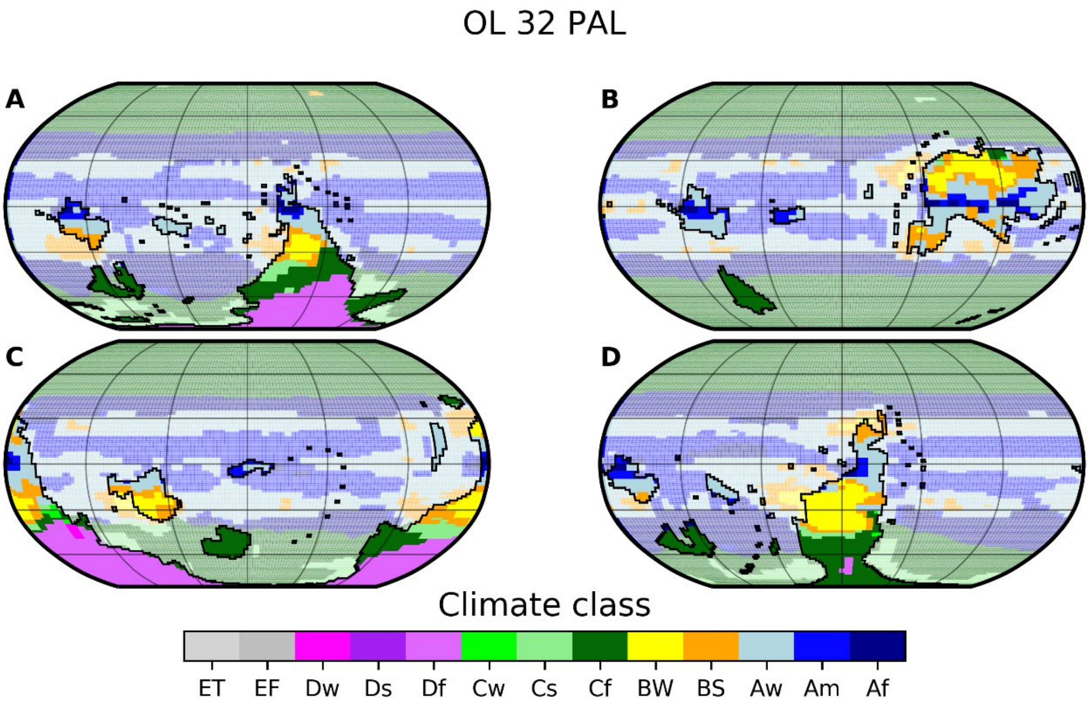

Supplementary Figure 32. Modelled Köppen–Geiger climate classes for a low obliquity orbit at 32 PAL CO_2. See Supplementary Table 5 for climate classes and Supplementary Table 2 for orbital parameters.

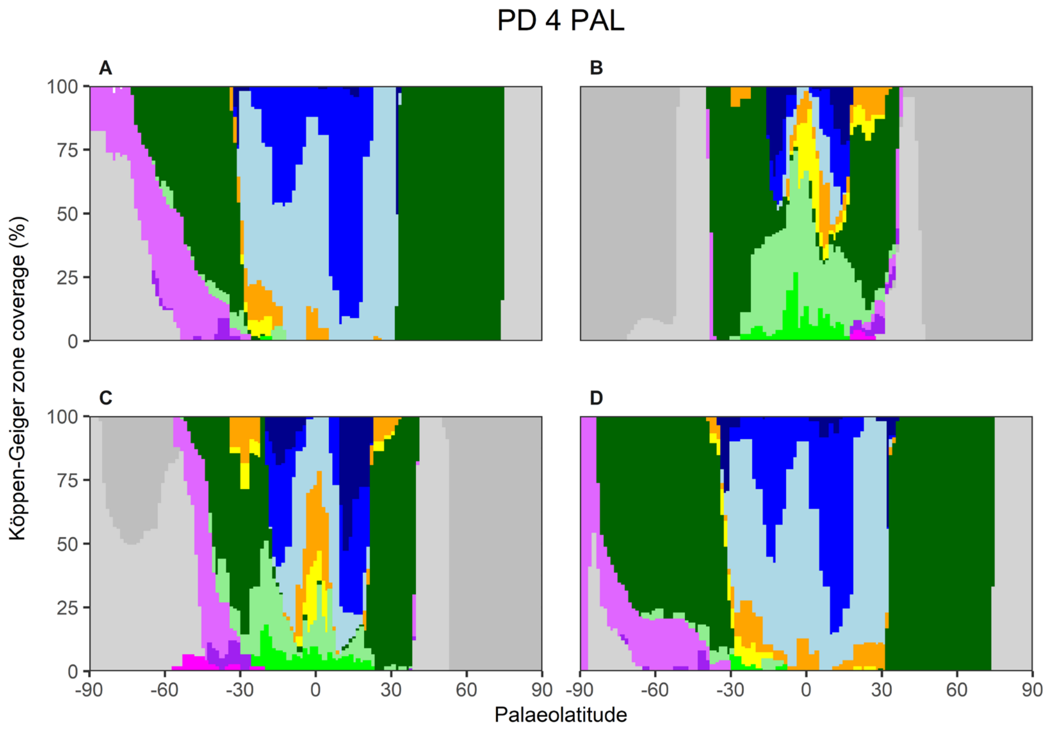

Supplementary Figure 33. Zonal frequency of modelled Köppen–Geiger climate classes for each configuration for a present day orbit at 4 PAL CO_2. See Supplementary Table 5 for climate classes and Supplementary Table 2 for orbital parameters.

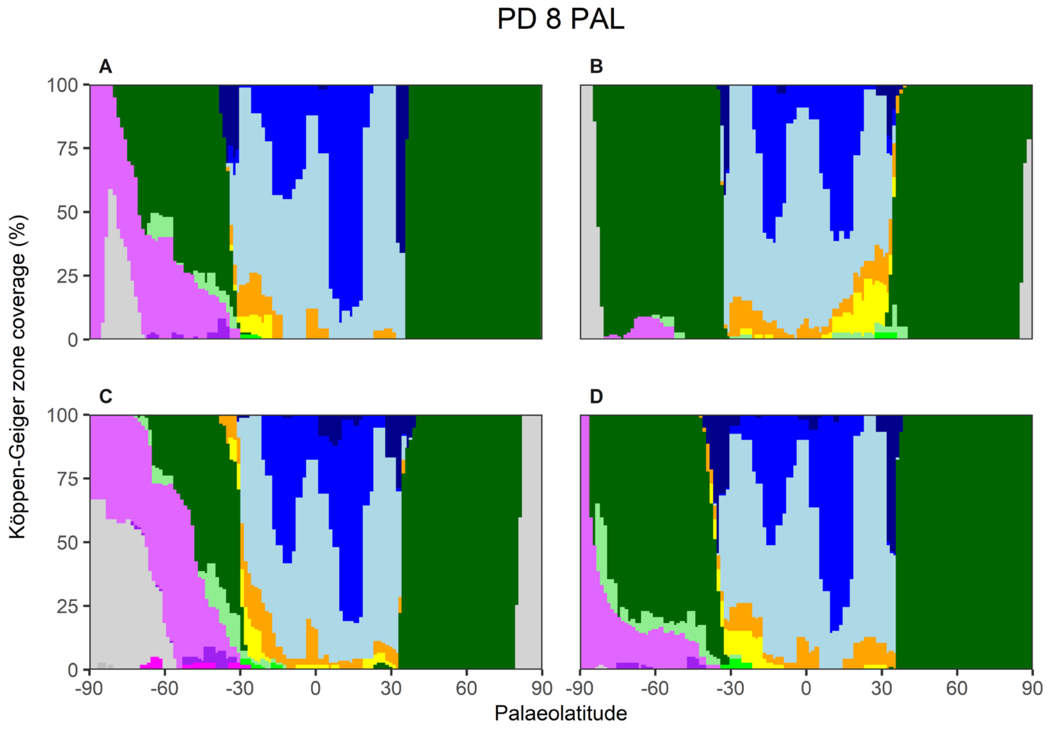

Supplementary Figure 34. Zonal frequency of modelled Köppen–Geiger climate classes for each configuration for a present day orbit at 8 PAL CO_2. See Supplementary Table 5 for climate classes and Supplementary Table 2 for orbital parameters.

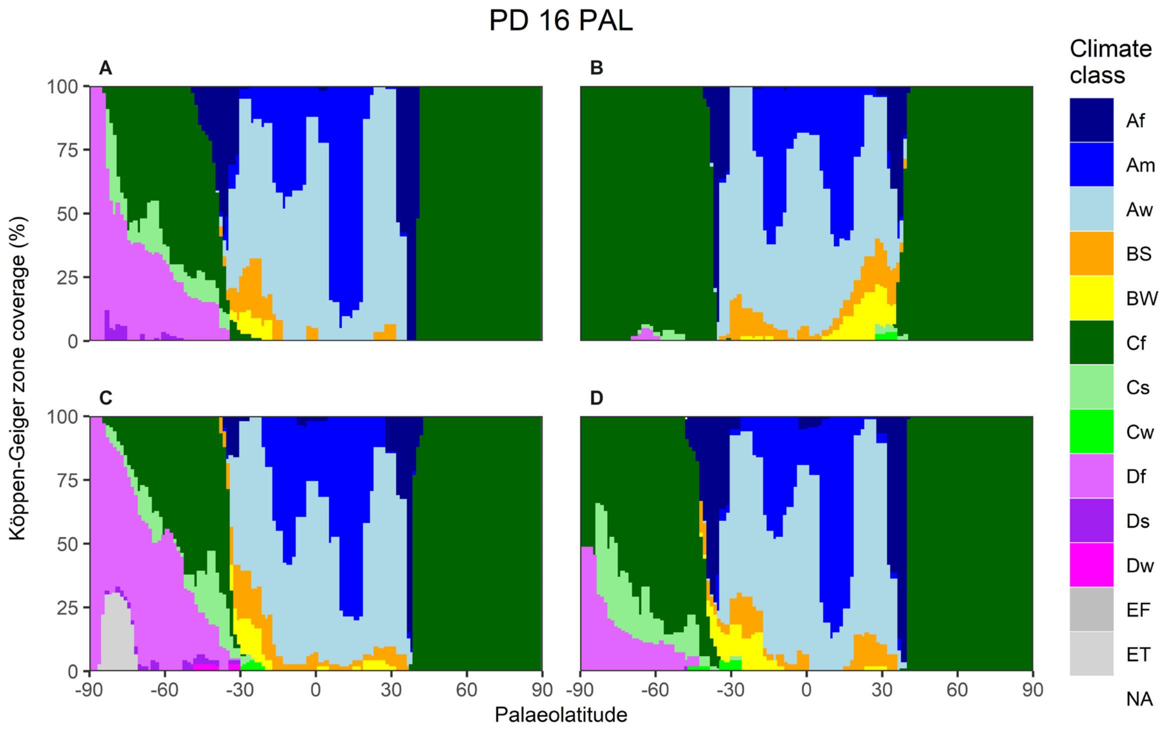

Supplementary Figure 35. Zonal frequency of modelled Köppen–Geiger climate classes for each configuration for a present day orbit at 16 PAL CO_2. See Supplementary Table 5 for climate classes and Supplementary Table 2 for orbital parameters.

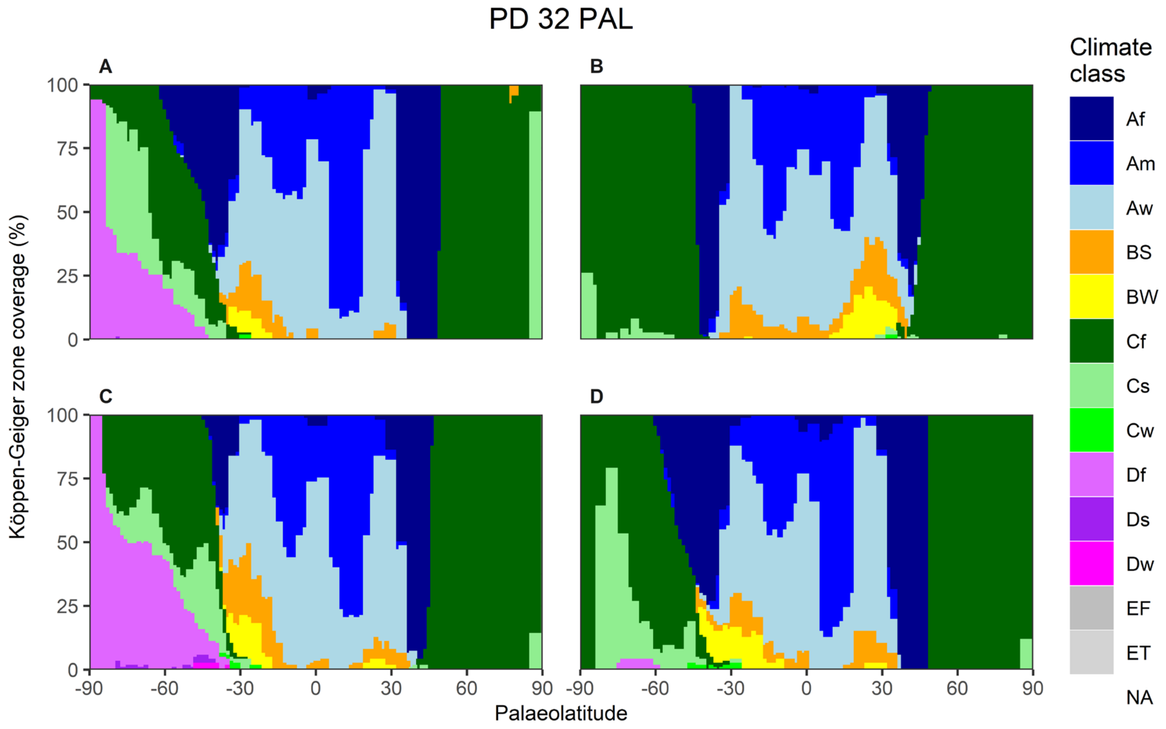

Supplementary Figure 36. Zonal frequency of modelled Köppen–Geiger climate classes for each configuration for a present day orbit at 32 PAL CO_2. See Supplementary Table 5 for climate classes and Supplementary Table 2 for orbital parameters.

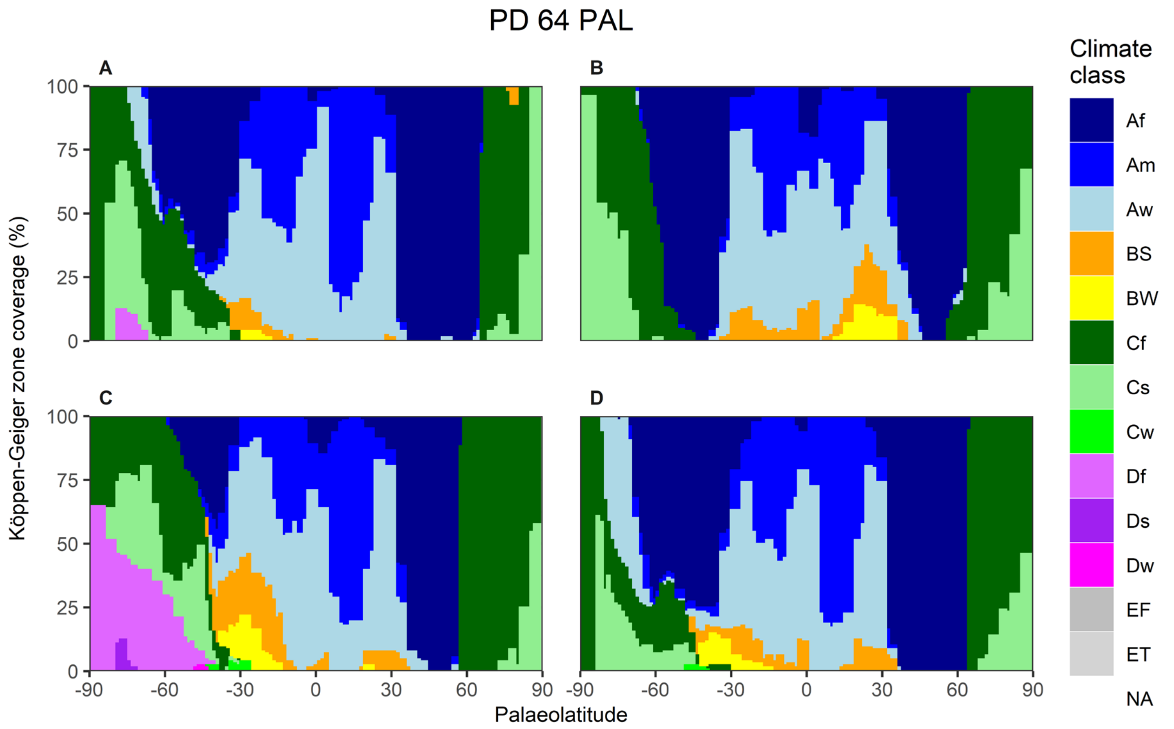

Supplementary Figure 37. Zonal frequency of modelled Köppen–Geiger climate classes for each configuration for a present day orbit at 64 PAL CO_2. See Supplementary Table 5 for climate classes and Supplementary Table 2 for orbital parameters.

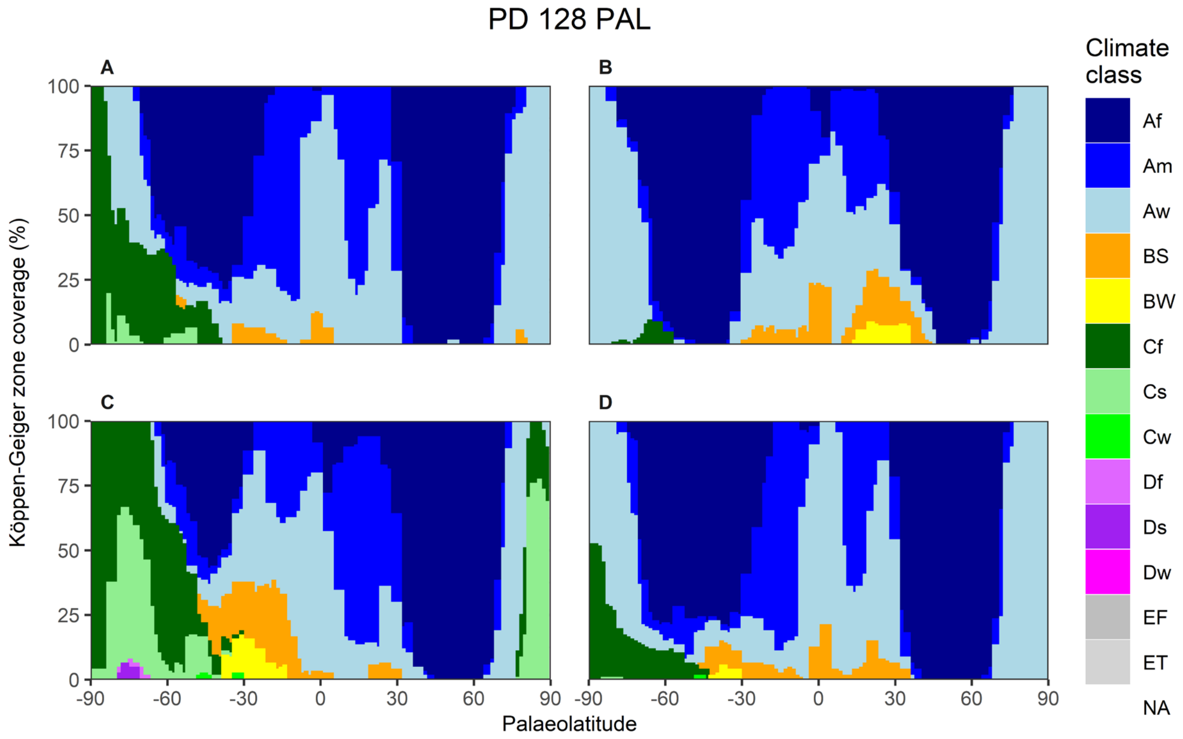

Supplementary Figure 38. Zonal frequency of modelled Köppen–Geiger climate classes for each configuration for a present day orbit at 128 PAL CO_2. See Supplementary Table 5 for climate classes and Supplementary Table 2 for orbital parameters.

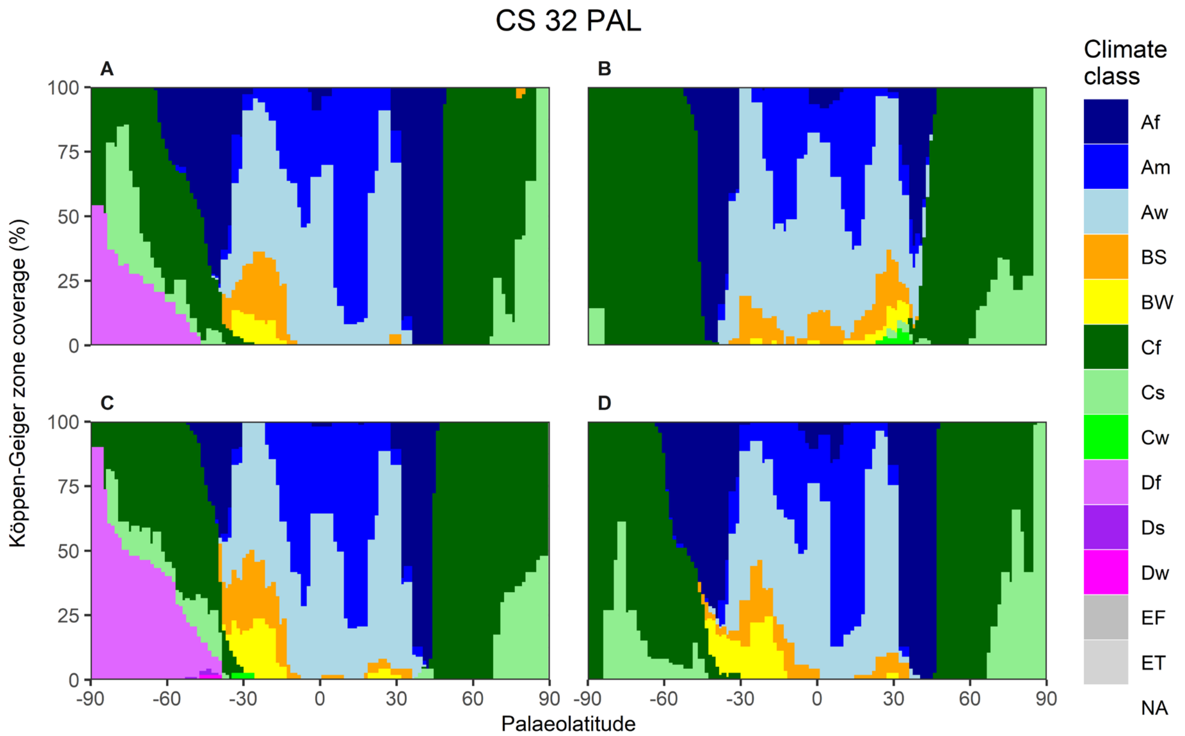

Supplementary Figure 39. Zonal frequency of modelled Köppen–Geiger climate classes for each configuration for a cold (austral) summer orbit at 32 PAL CO_2. See Supplementary Table 5 for climate classes and Supplementary Table 2 for orbital parameters.

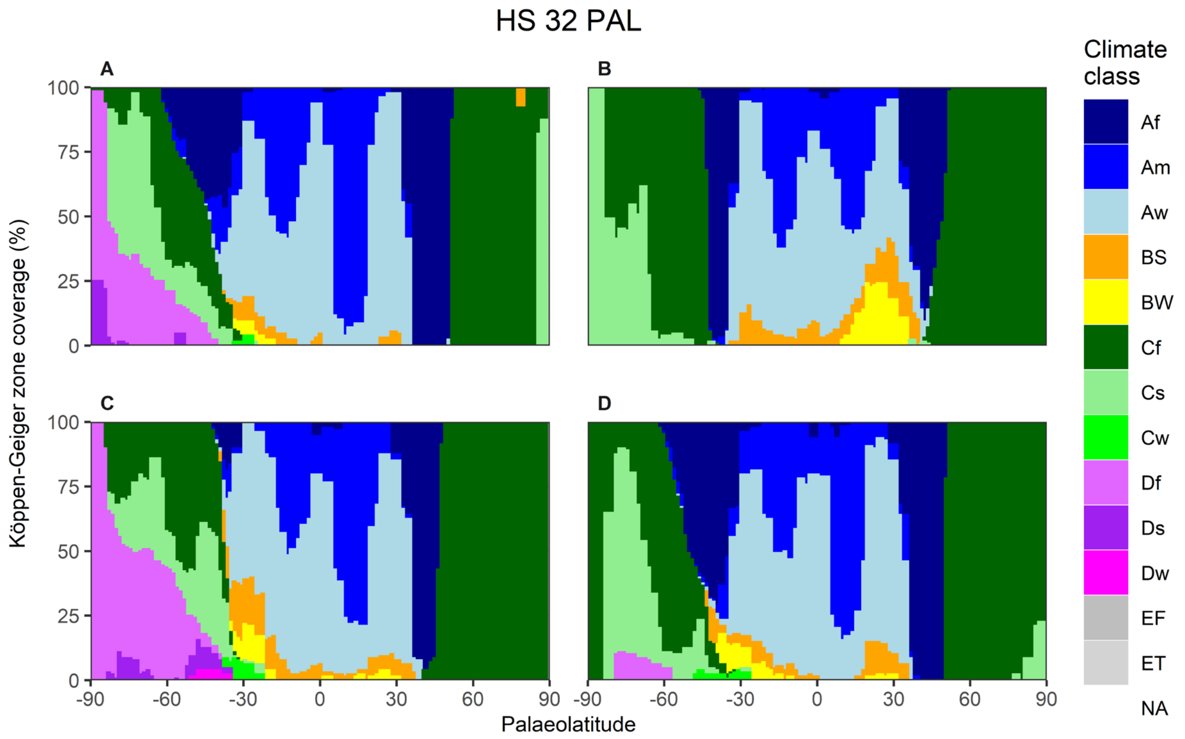

Supplementary Figure 40. Zonal frequency of modelled Köppen–Geiger climate classes for each configuration for a warm (austral) summer orbit at 32 PAL CO_2. See Supplementary Table 5 for climate classes and Supplementary Table 2 for orbital parameters.

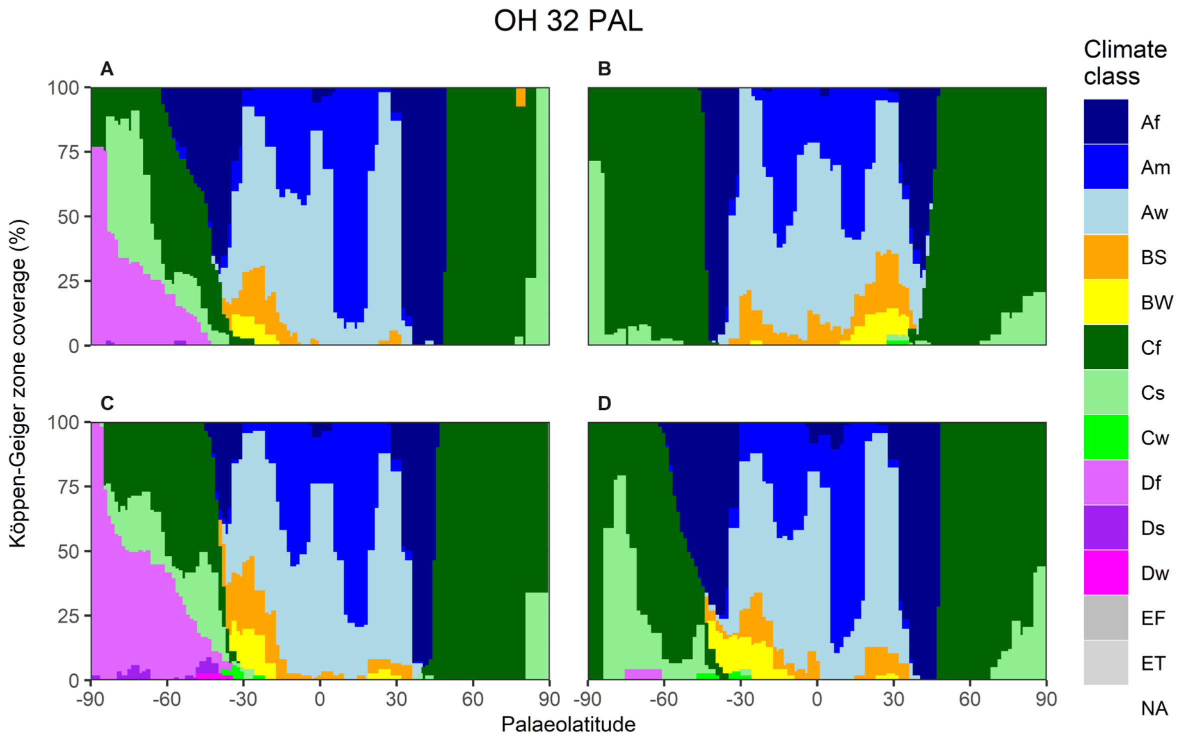

Supplementary Figure 41. Zonal frequency of modelled Köppen–Geiger climate classes for each configuration for a high obliquity orbit at 32 PAL CO_2. See Supplementary Table 5 for climate classes and Supplementary Table 2 for orbital parameters.

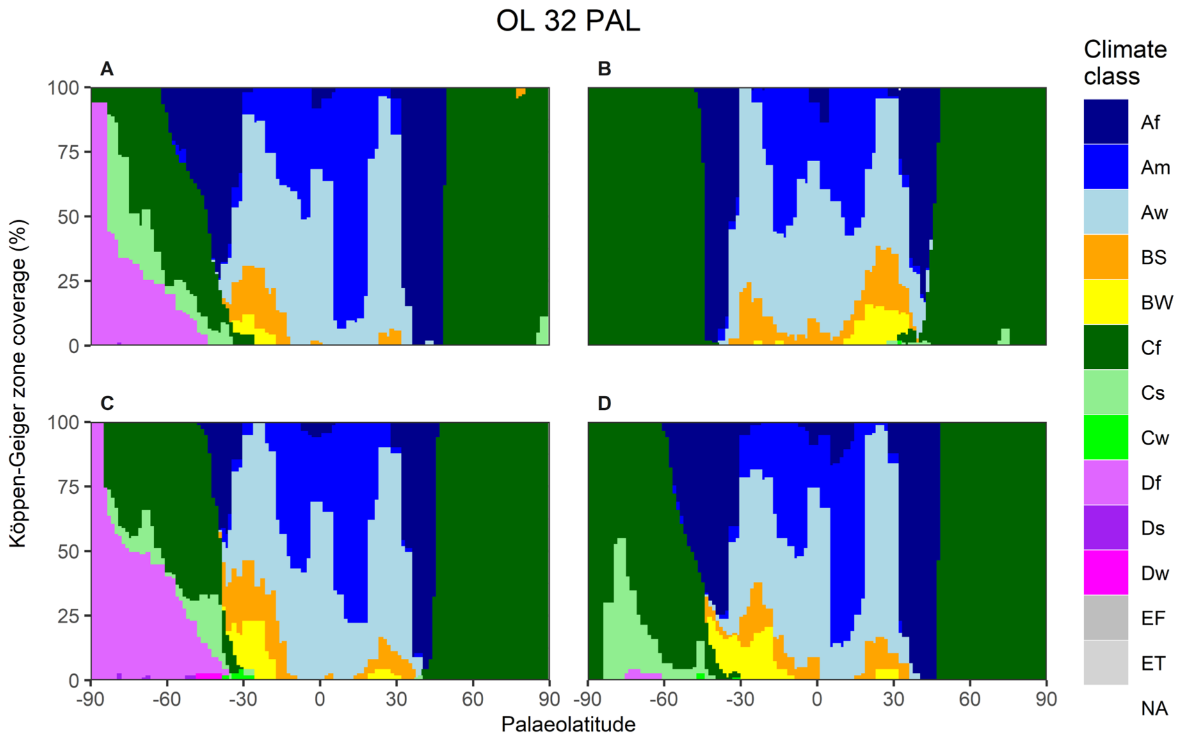

Supplementary Figure 42. Zonal frequency of modelled Köppen–Geiger climate classes for each configuration for a low obliquity orbit at 32 PAL CO_2. See Supplementary Table 5 for climate classes and Supplementary Table 2 for orbital parameters.

Supplementary Notes 4: Data–Model comparison

Supplementary methods. The mean number of grid cells within each radius over which palaeogeographic uncertainty was calculated for each continental configuration are presented in Supplementary Table 7.

Supplementary results. Data–model agreement scores accounting for palaeogeographic uncertainty, calculated using the code supplied in Supplementary Data 4, are presented in Supplementary Figure 43 to Supplementary Figure 47. The results of two-way analysis of variance (ANOVA) and Tukey's tests on the 32 PAL pCO_2 simulations only (Supplementary Table 8) and on all simulations (Supplementary Table 9). Supplementary Figure 48 present a the results of a sensitivity analysis. .

Supplementary Table 7. Mean number of grid cells for each configuration at each analysed radius. Total 16384 cells (128x128) in the FOAM GCM grid.

	Mean number of matched grid cells for each data set within given radius							
	Lower Cambrian				Series 2			
Configuration	200 km	250 km	500 km	1000 km	200 km	250 km	500 km	1000 km
A	3.94	6.38	25.7	104	3.90	6.25	25.7	104
B	2.86	4.21	17.2	69.8	2.89	4.06	16.8	68.3
C	3.48	5.26	21.4	85.9	3.50	5.33	21.8	87.3
D	3.12	4.87	19.5	78.1	2.99	4.61	18.8	75.3

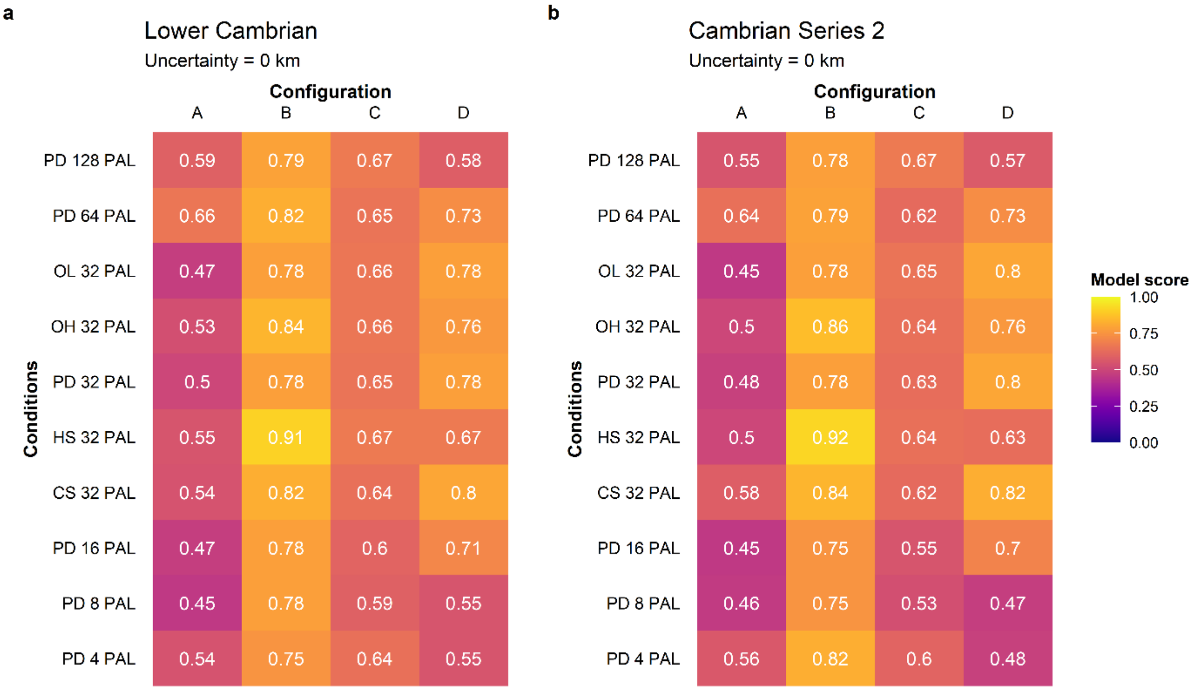

Supplementary Figure 43. Exact data–model agreement scores for all simulations, with no accounting for palaeogeographic uncertainty.

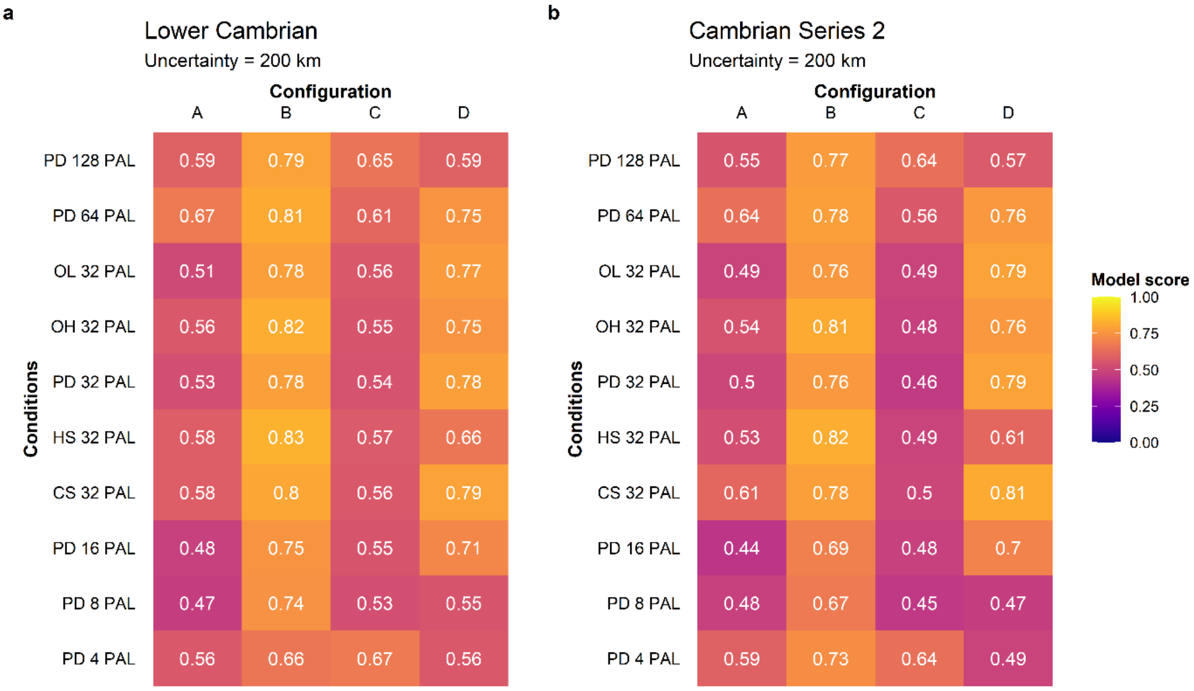

Supplementary Figure 44. Data–model agreement scores for all simulations with scores accounting for palaeogeographic uncertainty with a 200 km radius.

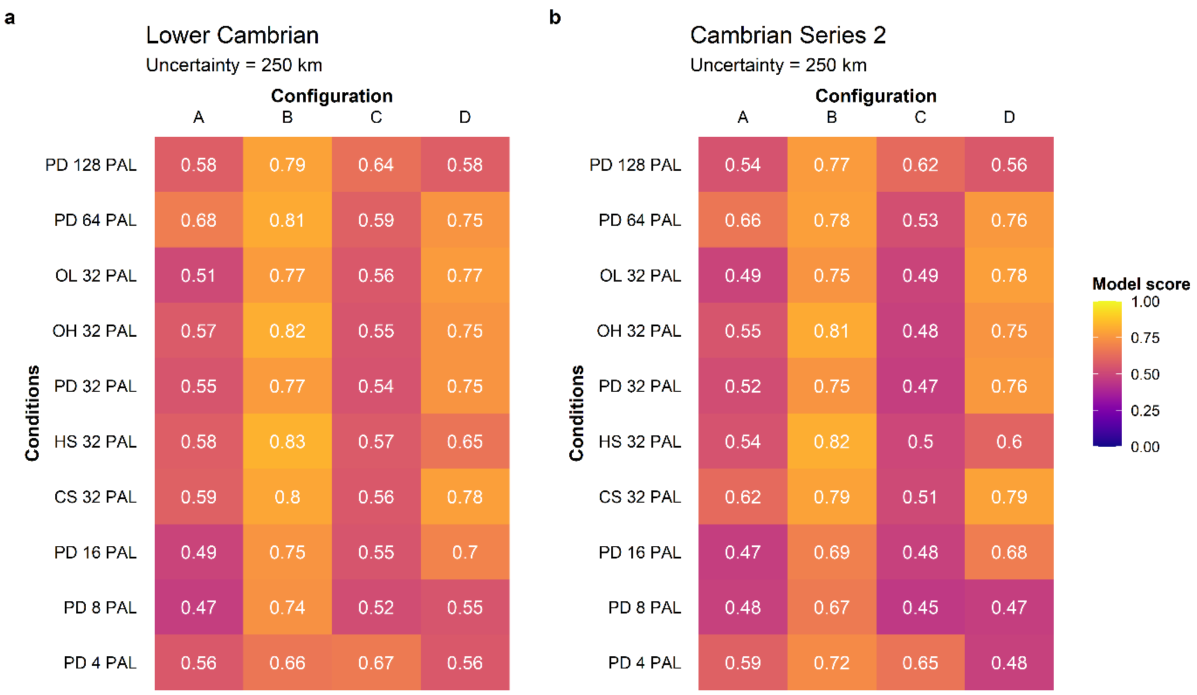

Supplementary Figure 45. Data–model agreement scores for all simulations with scores accounting for palaeogeographic uncertainty with a 250 km radius.

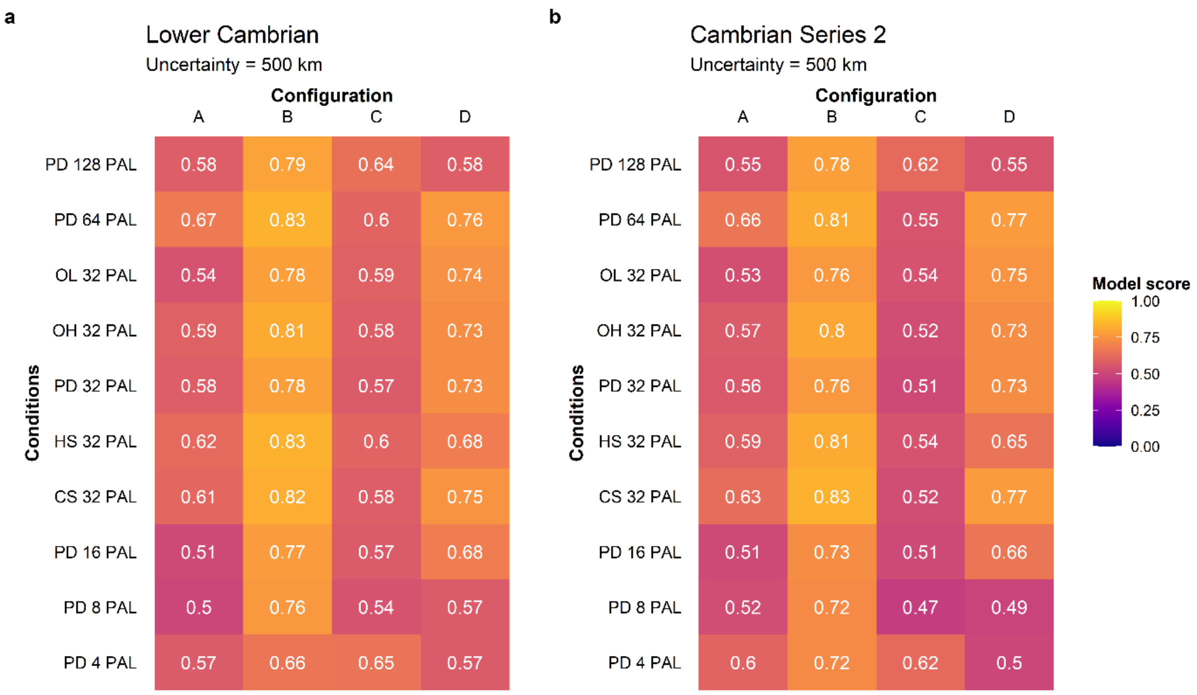

Supplementary Figure 46. Data–model agreement scores for all simulations with scores accounting for palaeogeographic uncertainty with a 500 km radius.

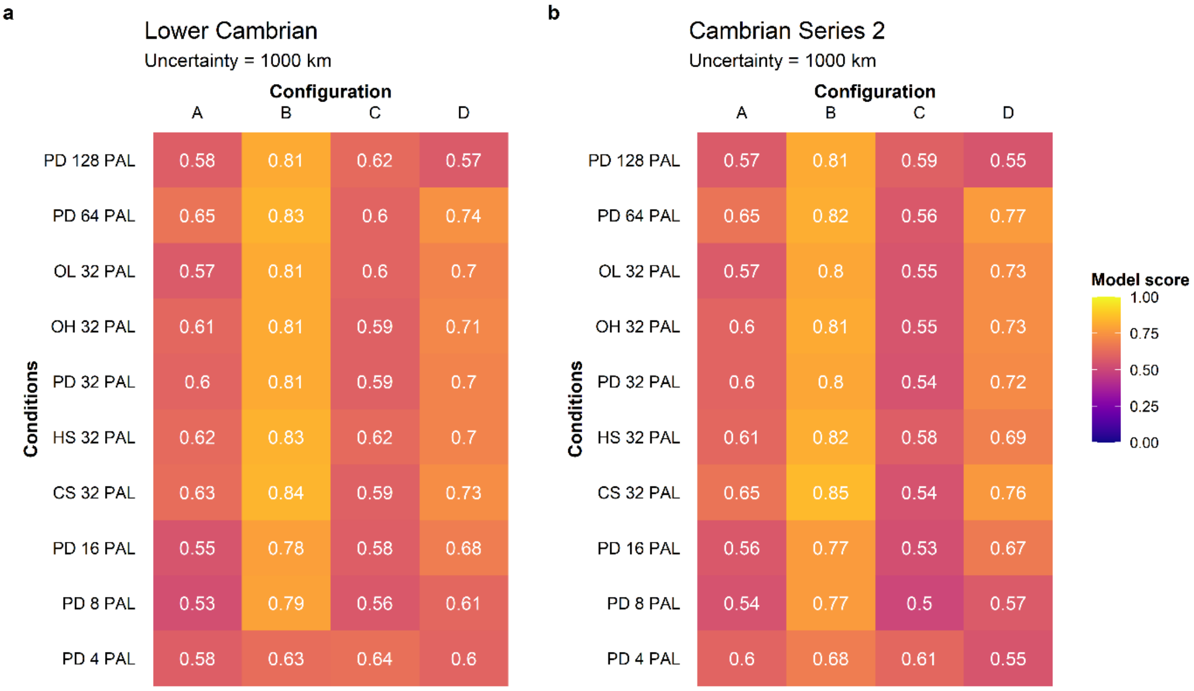

Supplementary Figure 47. Data–model agreement scores for all simulations with scores accounting for palaeogeographic uncertainty with a 1000 km radius.

Supplementary Table 8. Summary of two-way analysis of variance (ANOVA) and Tukey tests comparing the similarity of data–model agreement scores grouped by base configuration for 32 PAL CO2 simulations for all orbital parameters.

Test	Lower Cambrian scores accounting for uncertainty at:					Cambrian Series 2 scores accounting for uncertainty at:				
	0 km	200 km	250 km	500 km	1000 km	0 km	200 km	250 km	500 km	1000 km
ANOVA										
F(3,16)	53.59	77.26	66.97	99.13	202.79	36.35	46.94	42.42	67.52	130.78
p	1.44E-08	9.80E-10	2.83E-09	1.51E-10	6.14E-13	2.26E-07	3.74E-08	7.67E-08	2.66E-09	1.82E-11
Tukey[a]										
p(A, B)	1.30E-08	1.21E-08	3.53E-08	1.14E-09	3.66E-12	2.32E-07	2.92E-06	4.89E-06	1.40E-07	8.92E-10
p(A, C)	3.55E-04	**9.97E-01**	**9.98E-01**	**9.94E-01**	**8.65E-01**	6.37E-03	**4.10E-01**	**3.39E-01**	**1.33E-01**	9.94E-03
p(A, D)	4.31E-07	3.22E-07	1.63E-06	6.61E-07	1.85E-07	6.34E-06	1.80E-05	7.25E-05	1.67E-05	2.34E-06
p(B, C)	3.50E-05	1.53E-08	2.79E-08	8.63E-10	2.20E-12	1.42E-04	2.61E-07	3.35E-07	7.27E-09	2.65E-11
p(B, D)	**7.77E-02**	**1.00E-01**	**6.73E-02**	5.85E-04	5.01E-08	**1.80E-01**	**7.05E-01**	**4.37E-01**	3.31E-02	7.87E-05
p(C, D)	5.78E-03	4.26E-07	1.22E-06	4.49E-07	6.46E-08	1.02E-02	1.31E-06	3.56E-06	4.05E-07	1.40E-08

[a]*p(Configuration 1, Configuration 2)*

Grey text: significant difference at the 95% confidence level (< 5.00E-02).

Bold text: no significant difference at the 95% confidence level (> 5.00E-02).

Supplementary Table 9. Summary of two-way analysis of variance (ANOVA) and Tukey tests comparing the similarity of data–model agreement scores grouped by base configuration for all simulations.

Test	Lower Cambrian scores accounting for uncertainty at:					Cambrian Series 2 scores accounting for uncertainty at:				
	0 km	200 km	250 km	500 km	1000 km	0 km	200 km	250 km	500 km	1000 km
ANOVA										
F(3,36)	31.65	25.00	24.70	29.47	39.06	22.49	18.54	18.18	23.56	37.03
p	3.39E-10	6.41E-09	7.42E-09	8.42E-10	2.06E-11	2.23E-08	1.93E-07	2.37E-07	1.30E-08	4.25E-11
Tukey[a]										
p(A, B)	1.09E-10	2.92E-08	4.13E-08	3.73E-09	1.37E-10	9.54E-09	5.76E-06	8.53E-06	6.34E-07	6.94E-09
p(A, C)	1.93E-03	**8.10E-01**	**9.34E-01**	**9.29E-01**	**9.87E-01**	4.83E-02	**9.63E-01**	**8.67E-01**	**7.17E-01**	**3.69E-01**
p(A, D)	1.30E-05	1.97E-04	5.40E-04	1.14E-03	2.43E-03	5.25E-04	3.92E-03	1.37E-02	3.02E-02	1.31E-02
p(B, C)	1.17E-05	4.20E-07	2.43E-07	2.19E-08	3.40E-10	3.38E-05	1.32E-06	8.07E-07	2.66E-08	6.94E-11
p(B, D)	1.75E-03	3.00E-02	1.73E-02	9.00E-04	1.17E-05	4.77E-03	**1.44E-01**	**7.14E-02**	3.82E-03	1.22E-04
p(C, D)	**3.51E-01**	2.64E-03	2.96E-03	6.23E-03	6.05E-03	**3.46E-01**	1.00E-03	1.68E-03	1.82E-03	1.22E-04

[a]*p(Configuration 1, Configuration 2)*

Grey text: significant difference at the 95% confidence level (< 5.00E-02).

Bold text: no significant difference at the 95% confidence level (> 5.00E-02).

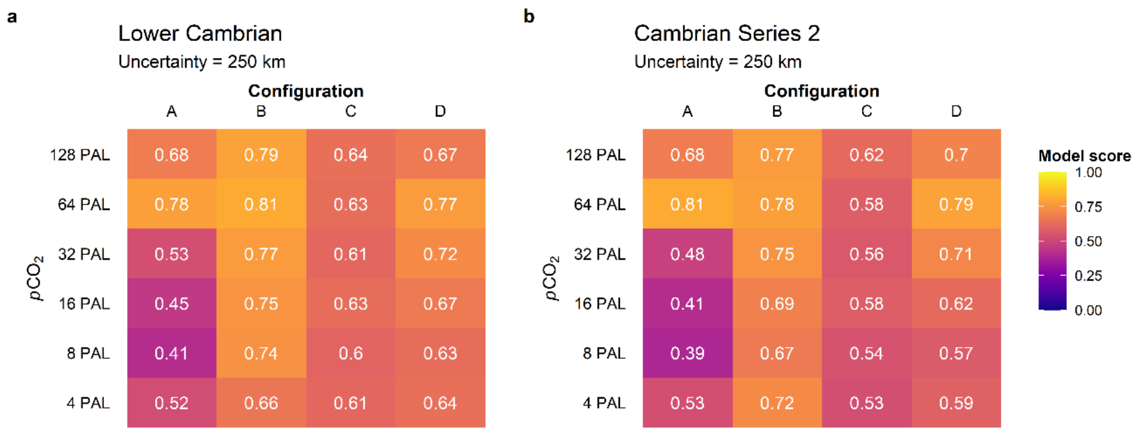

Supplementary Figure 48. Data–model agreement for all present day orbital configuration simulations scored against geological data excluding the North China palaeocontinent accounting for palaeogeographic uncertainty with a 250 km radius. The North China palaeocontinent is not included in the original reconstructions of configuration B[14,15], despite having extensive lower Cambrian deposits[e.g. 16]. Sensitivity analyses show that our results are robust to the systematic removal of all geological data from the North China palaeocontinent; whilst the numerical scores change, the overall patterns remain.

Supplementary References

1. Zhang, X. *et al.* Challenges in defining the base of Cambrian Series 2 and Stage 3. *Earth-Science Reviews* **172**, 124–139 (2017).
2. Landing, E., Schmitz, M. D., Geyer, G., Trayler, R. B. & Bowring, S. A. Precise early Cambrian U–Pb zircon dates bracket the oldest trilobites and archaeocyaths in Moroccan West Gondwana. *Geological Magazine* (2020) doi:10.1017/S0016756820000369.
3. Sundberg, F. A. *et al.* Asynchronous trilobite extinctions at the early to middle Cambrian transition. *Geology* **48**, 441–445 (2020).
4. Pohl, A., Donnadieu, Y., Le Hir, G., Buoncristiani, J.-F. & Vennin, E. Effect of the Ordovician paleogeography on the (in)stability of the climate. *Clim. Past* **10**, 2053–2066 (2014).
5. Lenton, T. M., Daines, S. J. & Mills, B. J. W. COPSE reloaded: An improved model of biogeochemical cycling over Phanerozoic time. *Earth-Science Reviews* **178**, 1–28 (2018).
6. Royer, D. L., Donnadieu, Y., Park, J., Kowalczyk, J. & Goddéris, Y. Error analysis of CO2 and O2 estimates from the long-term geochemical model GEOCARBSULF. *Am J Sci* **314**, 1259–1283 (2014).
7. Krause, A. J. *et al.* Stepwise oxygenation of the Paleozoic atmosphere. *Nature Communications* **9**, 4081 (2018).
8. Goddéris, Y., Donnadieu, Y., Le Hir, G., Lefebvre, V. & Nardin, E. The role of palaeogeography in the Phanerozoic history of atmospheric CO2 and climate. *Earth-Science Reviews* **128**, 122–138 (2014).
9. Locarnini, R. A. *et al. World Ocean Atlas 2013, Volume 1: Temperature*. (2013).
10. Nguyen, K. D. T. *et al.* Upper Temperature Limits of Tropical Marine Ectotherms: Global Warming Implications. *PLOS ONE* **6**, e29340 (2011).
11. NASA. World of Change: Global Temperatures. https://earthobservatory.nasa.gov/WorldOfChange/decadaltemp.php (2010).

12. Pendergrass, A., Wang, J.-J. & National Center for Atmospheric Research Staff. GPCP (Monthly): Global Precipitation Climatology Project. *The Climate Data Guide* https://climatedataguide.ucar.edu/climate-data/gpcp-monthly-global-precipitation-climatology-project (2018).
13. Peel, M. C., Finlayson, B. L. & McMahon, T. A. Updated world map of the Köppen-Geiger climate classification. *Hydrol. Earth Syst. Sci.* **11**, 1633–1644 (2007).
14. Landing, E., Geyer, G. & Brasier, M. D. Cambrian Evolutionary Radiation: context, correlation, and chronostratigraphy—overcoming deficiencies of the first appearance datum (FAD) concept. *Earth-Science Reviews* **123**, 133–172 (2013).
15. Landing, E., Westrop, S. R. & Bowring, S. A. Reconstructing the Avalonia palaeocontinent in the Cambrian: A 519 Ma caliche in South Wales and transcontinental middle Terreneuvian sandstones. *Geological Magazine* **150**, 1022–1046 (2013).
16. Chough, S. K. *et al.* Cambrian stratigraphy of the North China Platform: revisiting principal sections in Shandong Province, China. *Geosci J* **14**, 235–268 (2010).
