# Supplementary material for: Quantitative comparison of geological data and model simulations constrains early Cambrian geography and climate
Source: Nat Commun. 2021 Jun 23;12:3868. doi: 10.1038/s41467-021-24141-5 (PMC8222365; doi:10.1038/s41467-021-24141-5)
Supplement: Supplementary file 3 — Description of Additional Supplementary Files [file 41467_2021_24141_MOESM3_ESM.pdf]

Description of Additional Supplementary Files

File name: Supplementary Data 1.
Description: Spreadsheet of lower Cambrian climatically sensitive lithologies; available as an Excel file with three sheets, including explanation and references, and as a csv file. Available on the Zenodo data repository: http://doi.org/10.5281/zenodo.4506617.

File name: Supplementary Data 2.
Description: The FOAM NetCDF output files produced for this work. Available on the Zenodo data repository: http://doi.org/10.5281/zenodo.4506617.

File name: Supplementary Code 1.
Description: R script necessary to convert temperature and precipitation data in NetCDF files to Köppen–Geiger climate classes. Available on the Zenodo data repository: http://doi.org/10.5281/zenodo.4506617.

File name: Supplementary Code 2.
Description: R script to re-run the data–model comparison analyses used in the manuscript. Available on the Zenodo data repository: http://doi.org/10.5281/zenodo.4506617.
